# Multifactorial genetic divergence processes drive the onset of speciation in an Amazonian fish

**DOI:** 10.1371/journal.pone.0189349

**Published:** 2017-12-20

**Authors:** Luiz Jardim de Queiroz, Gislene Torrente-Vilara, Claudio Quilodran, Carolina Rodrigues da Costa Doria, Juan I. Montoya-Burgos

**Affiliations:** 1 Department of Genetics and Evolution, University of Geneva, Geneva, Switzerland; 2 Department of Marine Sciences, Universidade Federal de São Paulo, Campus Baixada Santista, Santos/SP, Brazil; 3 Institute of Genetics and Genomics in Geneva (iGE3), University of Geneva, Switzerland; 4 Department of Biology, Universidade Federal de Rondônia, Porto Velho/RO, Brazil; National Cheng Kung University, TAIWAN

## Abstract

Understanding the processes that drive population genetic divergence in the Amazon is challenging because of the vast scale, the environmental richness and the outstanding biodiversity of the region. We addressed this issue by determining the genetic structure of the widespread Amazonian common sardine fish *Triportheus albus* (Characidae). We then examined the influence, on this species, of all previously proposed population-structuring factors, including isolation-by-distance, isolation-by-barrier (the Teotônio Falls) and isolation-by-environment using variables that describe floodplain and water characteristics. The population genetics analyses revealed an unusually strong structure with three geographical groups: Negro/Tapajós rivers, Lower Madeira/Central Amazon, and Upper Madeira. Distance-based redundancy analyses showed that the optimal model for explaining the extreme genetic structure contains all proposed structuring factors and accounts for up to 70% of the genetic structure. We further quantified the contribution of each factor via a variance-partitioning analysis. Our results demonstrate that multiple factors, often proposed as individual drivers of population divergence, have acted in conjunction to divide *T*. *albus* into three genetic lineages. Because the conjunction of multiple long-standing population-structuring processes may lead to population reproductive isolation, that is, the onset of speciation, we suggest that the multifactorial population-structuring processes highlighted in this study could account for the high speciation rate characterising the Amazon Basin.

## Introduction

The Amazon Basin is the largest freshwater system and one of the most species-rich basins in the world. This region harbours elevated intraspecific diversity in many taxa [1,2], including fish [3]. Unravelling the processes that have generated such inter- and intraspecific diversity has been a focus of evolutionary biologists for more than a century [4] and remains challenging [5]. However, the accumulation of diversity-related knowledge and recent advances in landscape genetic approaches have provided insights on this issue, and several adaptive and neutral evolutionary processes have been suggested to explain how fish populations have diverged.

The most intuitive process explaining Amazonian fish population divergence is the reduction in gene flow among the geographically dispersed populations occurring along the extensive and distant Amazonian tributaries. This mechanism of divergence has been suggested to be the cause of the so-called pattern of isolation-by-distance (IBD). The underlying process of IBD predicts that the genetic similarity among populations tends to decline with increasing geographical distance [6]. A strong pattern of IBD has been found in Amazonian fish populations, and its underlying cause is thought to have a substantial effect on population structure [7–9], which is particularly evident in widespread species that display limited dispersal ability in the enormous extent of the Amazonian river network [10].

In complex and heterogeneous environments, factors other than geographical distance may affect population structure. Spatial differences in environmental features may result in different local selective pressures that trigger adaptive responses and drive the evolution of traits that are ecologically relevant for local conditions. Individuals with adaptations to local habitats might present low fitness in locations with non-equivalent habitats, whereas they may perform well in locations with equivalent habitats regardless of the geographical distance between these locations. Hence, less gene flow is expected among environmentally dissimilar locations than among similar locations, and this process may underlie patterns of isolation-by-environment (IBE) [11].

Annual fluctuations in river water levels lead to inundation of riparian vegetation during rising periods forming floodplains of varying sizes, and this process represents a conspicuous characteristic of the Amazon Basin that might lead to patterns of IBE. For many fish species, the extension of the floodplains can determine the carrying capacity and the size of local populations by modifying food abundance and availability, spawning and offspring success [12]. Flooded riparian forests are a determinant of the subsistence of many different species. Herbivorous, omnivorous and insectivorous fishes have undergone adaptations to benefit from the access to the forest during the rainy season, where they forage on forest fruits, seeds and invertebrates [13]. Moreover, many fish species undertake lateral migrations from the main channel into the floodplains to optimize their development, growth, and reproduction [14]. Hence, in the Amazon, fishes have locally evolved to take advantage of the temporary habitats that are periodically accessible [15]. Thus, local floodplain sizes and vegetation compositions may be a source of divergent adaptation among the populations of fishes inhabiting river sections with different characteristics, thereby generating patterns of IBE.

The Amazon Basin displays another peculiar feature that contributes to its landscape heterogeneity: the water quality, which is most often referred to as the water colour. Three major types of water colour are recognized: blackwater, clearwater and whitewater [16]. Most blackwater rivers drain pre-Cambrian cratonic areas of the Guyana Shield, carry low levels of dissolved solids, display low turbidity and convey enormous concentrations of organic matter (humic acids), thus resulting in highly acidic (pH ~4) and dark but transparent waters. A typical blackwater river is the Negro River. Clearwater is less acidic than blackwater (pH ~6.5) because of the reduced concentration of humic acids, but it also has low conductivity, is highly transparent, and presents a greenish background colour. Clearwater rivers also drain ancient uplands (Guyana and Brazilian Shields), and typical examples are the Tapajós and Xingu rivers. Whitewater, however, has a neutral pH, is highly turbid, has a high concentration of dissolved solids and presents a whitish load of sand and mud particles coming from the Andean piedmont, which is the source area of the headwaters of whitewater rivers [16,17]. Water colour has been noted as an important determinant of aquatic communities by delimiting the distribution and controlling the abundance of several aquatic organisms, especially fish [18–21]. However, the potential adaptive responses of Amazonian fishes to different water colours have been tested only recently [22–24], and although poorly understood, these responses reveal an important promoter of diversification [25].

In addition to the aforementioned population-structuring processes, landscape breaks may also contribute to population divergence via gene flow limitation. The pattern of population differentiation associated with landscape breaks is referred to as isolation-by-barrier (IBB). In the Amazon Basin, waterfalls and rapids are important geographical features that fragment the riverscape. These types of physical barriers may act as major obstacles to dispersal or migration for certain aquatic species [26], thereby leading to population structure or allopatric speciation [27].

The Madeira River is one of the main tributaries of the Amazon Basin and displays a 300-km stretch of waterfalls and rapids in the downstream border of its upper portion that partitions the river course. These rapids and waterfalls have likely played a historical role as filters or barriers to gene flow for many aquatic and semi-aquatic vertebrates [28–32]. However, between 2008 and 2011, two hydroelectric power plants, the Santo Antônio Dam (http://www.santoantonioenergia.com.br) and the Jirau Dam (http://www.energiasustentaveldobrasil.com.br) were constructed in this section of the Madeira River. The reservoirs modified the aquatic habitat and permanently flooded an important upstream area that hosted many of these rapids and waterfalls.

To date, most of the proposed processes driving population divergence in the Amazon and their derived patterns of isolation have been analysed individually. Here, we aimed to investigate the joint action of these processes as a possible explanation for the genetic population structure of a representative migratory Amazonian fish, *Triportheus albus* (Characidae), locally known as the common sardine. It is an ideal candidate because of its wide distribution, high abundance, ability to inhabit most riverine habitats including all water colour types and facility of capture [33,34]. Previous studies have indicated that this species is susceptible to some of the structuring processes suggested for Amazonian fishes [22]. For instance, patterns of IBD at moderate geographical scales (~100 km) and IBE have been documented in the Central Amazon, with the latter resulting from a possible adaptation to different water colours [22]. Moreover, similar to many Amazonian fishes, this species undertakes lateral migrations into various types of flooded plains to reach feeding areas during the season when the rivers rise, which might lead to patterns of IBE.

## Material and methods

### Ethics statement

The permissions to collect (83/2012) and to export (12BR008351/DF, 12BR009559/DF, 13BR011307/DF and 13BR011335/DF) the biological material was provided by the *Instituto Brasileiro do Meio Ambiente e dos Recursos Naturais Renováveis* (Ibama). In accordance with the Brazilian laws, and following the recommendations of the *Conselho Federal de Medicina Veterinária* (CFMV) and the *Conselho Nacional de Controle de Experimentação Animal* (CONCEA), the specimens, which were collected by the authors, were anaesthetised and euthanised with eugenol (50 mg/L^-1^).

### Study organism

The Amazonian common sardine, *T*. *albus*, is a member of the family Characidae [35], but sometimes referred as belonging to the Triportheidae [36]. It is a medium-sized species that can reach up to 23 cm of standard length [37], and shows a fast growth rate [38]. This species reaches sexual maturity at approximately one year of age (personal observations).

Medium–long migrations of up to 100 km for reproduction have been reported for *T*. *albus* [13,39]. Reproductive migration is synchronised with the flood pulse: every year, during the early rainy season, when the level of the rivers starts increasing, specimens living in tributaries form large schools that migrate downstream to spawn in the main channel of rivers [13]. Fertilized eggs drift further downstream and are transported into the floodplains, where larval fish find abundant shelter and food [40]. *T*. *albus* does not show any parental care, its fecundity is usually very high, with females producing more than 7000 oocytes in one unique reproductive cycle, spawned at once [38]. These reproductive characteristics and migratory behaviour are shared among many migratory fishes in the Amazon [39].

The dependence of *T*. *albus* on the floodplains goes beyond the importance for the first life stages, as multiple lateral migrations along the year, from the main channel to the floodplains, have been registered for adults [14]. These movements are related to the feeding habits since its omnivorous diet includes an important fraction of terrestrial invertebrates, seeds and fruits [41–43].

*Triportheus albus* is constantly present in the subsistence fisheries of many riverside human communities [44]. Furthermore, the reproductive migratory behaviour in large schools makes them an easy target for fishermen. On the other hand, the fast growth rate and the short time to reach sexual maturity make this species very resilient to anthropic actions, including intensive fishery. This could explain why, in the last few decades, the tonnage of fished *T*. *albus*, as well as many other medium-sized species with similar life histories, has substantially increased in Amazonia [33,34].

### Sampling and sequence data

**Fig 1:**
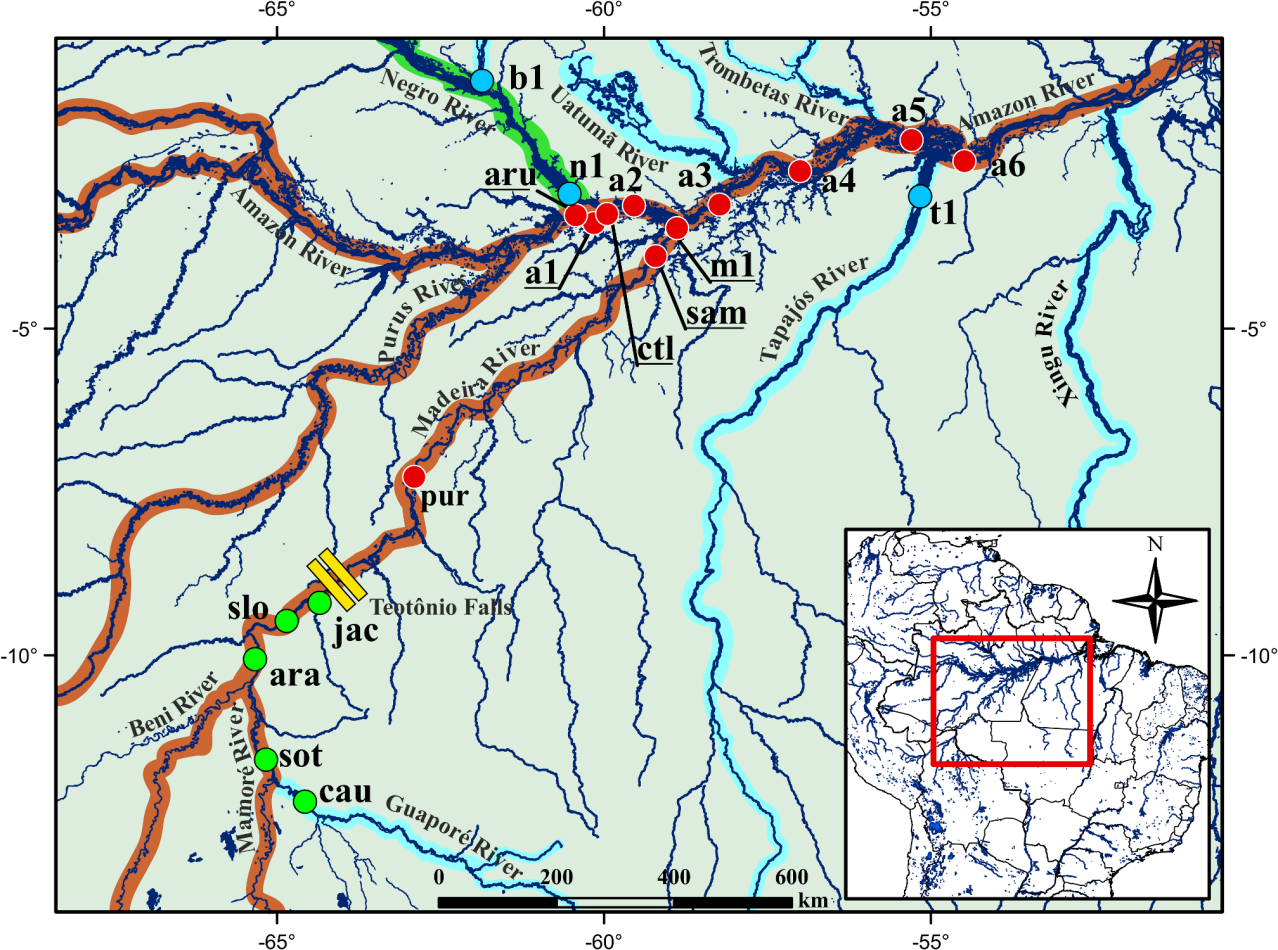
Study area, including the sampling localities in the whitewater (Madeira and Amazon rivers), blackwater (Negro River) and clearwater (Tapajós River) rivers. Localities represented by green dots correspond to the Upper Madeira, upstream of the Teotônio Falls; red dots include the Lower Madeira and the main channel of the Amazon River; blue dots are the localities in the Negro and Tapajós rivers. Samples from cau (Cautário River), sot (Sotério River), ara (Arara River), slo (São Lourenço River), jac (Jaciparaná River), pur (Puruzinho Lake), sam (Sampaio Lake), ctl (Catalão Lake), and aru (Ariaú channel) were obtained in the present study, whereas samples of the remaining localities were obtained from the study of [22]. Rivers with a brown shadow are the main whitewater rivers in the Amazon Basin, whereas black- and clearwater rivers are shaded in green and blue, respectively.

Sampling sites were chosen based on two main aspects: (1) the water colour of the river, including whitewater, blackwater and clearwater localities; and (2) the Teotônio Falls of the Madeira River, which is a major landscape break in Amazonia (Fig 1; Table 1). The number of localities per characteristic region is as follows: *(i)* three localities in the Negro and Tapajós rivers that represent non-adjacent blackwater and clearwater rivers, respectively; *(ii)* eleven localities in the Central Amazon and Lower Madeira downstream of the Teotônio Falls that harbour whitewater; and *(iii)* five localities in the Upper Madeira River upstream of the Teotônio Falls that are dominated by Andean-originated whitewater, excluding the Cautário River locality, which is a clearwater river [16]. A total of 222 samples of *T*. *albus* were analysed (Table 1). The fish were caught with gillnets, seine nets, cast nets and trawl nets. Tissue samples were stored in 80% ethanol, and DNA was extracted using the peqGold Tissue DNA kit (Peqlab Ltd., Darmstadt, Germany).

**Table 1:** Sampling localities and genetic diversity indices based on the ATPase 6 & 8 for *Triportheus albus*.

Site/Region	Lat.	Long.	n	h	s	hd	π
cau	-12.20	-64.59	9	4	9	0.583	0.0027
sot	-11.61	-65.23	18	3	2	0.216	0.0003
ara	-10.02	-65.31	3	1	0	–	–
slo	-9.36	-64.85	4	1	0	–	–
jac	-9.29	-64.40	32	7	7	0.345	0.0006
**Upper Madeira**			**66**	**11**	**15**	**0.307**	**0.0006**
pur	-7.37	-63.05	4	2	2	0.667	0.0017
sam	-3.86	-59.19	7	3	2	0.524	0.0007
m1	-3.47	-58.87	8	4	4	0.643	0.0015
aru	-3.18	-60.35	10	2	1	0.200	0.0003
a1	-3.35	-60.15	8	6	8	0.929	0.0030
ctl	-3.24	-59.95	21	6	9	0.429	0.0011
a2	-3.13	-59.54	20	4	3	0.284	0.0004
a3	-3.08	-58.22	17	7	9	0.596	0.0015
a4	-2.55	-57.03	3	1	0	–	–
a5	-2.17	-54.97	3	2	4	–	–
a6	-2.47	-54.50	5	3	4	0.700	0.0028
**Lower Madeira/Central Amazon**			**106**	**23**	**30**	**0.525**	**0.0014**
n1	-3.08	-60.25	9	6	5	0.889	0.0022
b1	-1.27	-61.85	20	7	8	0.816	0.0020
t1	-2.87	-55.16	21	5	4	0.581	0.0008
**Negro/Tapajós Rivers**			**50**	**13**	**14**	**0.798**	**0.0017**

Lat. = latitude; Long. = longitude; n = sample size; h = number of haplotypes; s = number of variable sites; hd = gene diversity; π = nucleotide diversity; Cautário River (cau), Sotério River (sot), Araras River (ara), São Lourenco River (slo), Jaciparaná River (jac), Puruzinho Lake (pur), Sampaio Lake (sam), Madeira River, near its mouth (m1), Ariaú Channel (aru), Amazonas River 1, 2, 3, 4, 5 and 6 (a1, a2, a3, a4, a5, a6, respectively), ctl (Catalão Lake), Negro River (n1), Branco River (b1) and Tapajós River (t1).

A 792-base pair (bp) fragment containing part of two adjacent mitochondrial genes, *ATPase synthase subunit six* and *subunit eight* (ATPase 6 & 8), was amplified for all of our *T*. *albus* samples, 11 samples of *T*. *auritus*, one of *T*. *culter* and two of *T*. *brachipomus* (accession numbers: MF188209– MF188231; S3 Table) using the primers ATP8.2 and CO3.2 [45]. This mtDNA fragment has already been demonstrated to be suitable for reconstructing the evolutionary history of Neotropical fishes [46], including *T*. *albus* populations in the Amazon Basin, showing similar evolutionary patterns when compared to the whole genome [22]. Hence, most of the analyses of this work were based on this mitochondrial marker only. However, we also amplified 1265 bp of the exon 3 of the nuclear gene encoding the *recombination activating protein 1* (RAG1; accession numbers MF188235–MF188240 and MF350633–MF350657; S4 Table) for a subsample of 37 individuals coming from the three areas (Upper Madeira, Lower Madeira/Central Amazon, and Negro/Tapajós rivers). This nuclear marker was used to corroborate the genetic structure obtained with the mitochondrial ATPase 6 & 8.

To assess if the individuals used in our analyses belong to a single species, we also sequenced 868 bp of the *cytochrome c oxidase subunit I* (COI). This marker was sequenced in a subsample composed of seven specimens from the Negro and Tapajós rivers, four from the Lower Madeira/Central Amazon, and six from the Upper Madeira (S3 Data). The pairwise genetic divergence rates were then calculated using the Kimura 2-parameters model (K2P) in MEGA 6.06 [47] based on the standard barcode region for fishes [48], which covers 647 bp of the region sequenced. For comparison purposes, we also calculated the genetic divergence between other well-characterised and recognised *Triportheus* species. COI sequences of *T*. *auritus* and *T*. *brachipomus* were generated in this study, whereas sequences of *T*. *angulatus*, *T*. *guentheri* and *T*. *nematurus* were obtained from the GenBank sequence database (all GenBank accession numbers are present in S5 Table).

The PCR conditions and primer sequences for the three markers are presented in the supporting information (S1 Appendix; S1 Table). The sequences were aligned manually using BioEdit 7.1.3.0 [49]. As ATPase 6 & 8, RAG1 and COI are protein-coding genes, we checked for any errors or unexpected stop codons in the sequences using the translate tool of the Bioinformatics Resource Portal (ExPASy) of the Swiss Institute of Bioinformatics (SIB) (http://web.expasy.org/translate/). The complete alignments are available in the Supporting Information (S1–S3 Data).

### Phylogenetic reconstruction, divergence time and haplotype network

To confirm the monophyly of our *T*. *albus* samples, a maximum likelihood (ML) phylogenetic reconstruction was performed using RAxML 7.2.8 [50] based on the mitochondrial ATPase 6 & 8. This analysis was conducted using a subsample of 24 sequences of *T*. *albus*, 12 sequences of *T*. *auritus*, one of *T*. *culter* and two of *T*. *brachipomus* (S1 Data). One sequence of *Brycon oligolepis* was also included to root the tree. PartitionFinder 2 [51] was used beforehand to identify the best partitioning scheme and the best model of molecular evolution among those implemented in RAxML. To do so, we used three *a priori* partitioning schemes: (i) no partitions, (ii) two partitions: codon positions 1 plus 2 together, and position 3 alone, or (iii) three partitions: codon position 1, codon position 2 and codon position 3. To compare the models of molecular evolution and partitioning schemes, we used the corrected Akaike information criterion (AICc). PartitionFinder identified the partitioning scheme “*(ii)*” as the best under the GTRGAMMAI model, which we used in the RAxML analysis. For the RAxML analysis, the GTRGAMMAI model was set to four gamma rate categories, and we ran 50 iterations in the search for the most likely tree using the rapid hill-climbing algorithm. A separated bootstrap analysis was run with 1000 replicates. The bootstrap values were plotted on the ML tree.

We inferred a time-calibrated tree to estimate the divergence time between the main lineages within *T*. *albus* based on the complete ATPase 6 & 8 dataset using BEAST 2.4.6 [52]. For this purpose, no outgroups were used (S1 Data). We used the partition scheme previously defined for the RAxML analysis, that is, two partitions, codon positions 1 plus 2 together, and codon position 3 alone. To estimate a phylogeny averaged over site models, we used bModelTest [53] as implemented in BEAST2 [52]. For this, we used the default transition/transversion split option. For the divergence time estimation, we assumed a lognormal relaxed clock based on the recommendations of [54], since the coefficient of variation of the clock model was bigger than 0.1. A lognormal prior for the substitution rate of ATPase 6 & 8 was set with a mean of 0.007 substitutions/site/My and a standard deviation of 0.001, according to a widely used and accepted estimation for ray-finned fish mitochondrial protein-coding genes [45]. In BEAST2, the options that are available as tree priors include those designed for species-level data (the pure-birth and birth–death priors) and those that are more suitable to describe the relationships among individuals of the same population/species (coalescence-based priors). As we are working at the population-level, we used a coalescence-based prior [55]. We chose the coalescent skyline prior over the other coalescence-based options to avoid constraining *a priori* the population size through time, as it is the case in the other two alternatives (coalescent constant and exponential population). For full details, see the XML file used to run the BEAST2 analysis (S4 Data). We ran three independent runs with 80 million generations each and sampled every 8000^th^ generations. We determined the burn-in (10%) and confirmed the convergence of parameters in the Markov chain Monte Carlo (MCMC) by examining the effective sample size values (ESS > 200) and likelihood plots using Tracer 1.6 (http://beast.bio.ed.ac.uk/Tracer). The potential scale reduction factor (PSRF) of the parameters was calculated using the package coda [56] in R environment [57], and the results were close to 1.0 indicating approximate convergence. Moreover, we confirmed that the topologies of the three runs converged on the same space by using AWTY [58] as implemented in the R Package RWTY [59]. Pseudo-ESS of the tree topologies [60] was higher than 3900 for all the runs, and the average split frequencies was below 0.0002. For the time-consuming RWTY analyses, we used 50% of the trees.

We used LogCombiner 2.4.6 (http://beast.bio.ed.ac.uk/logcombiner) to combine the runs. Then, TreeAnnotator 2.4.6 (http://beast.bio.ed.ac.uk/treeannotator) was used to produce the maximum clade credibility trees from the post-burn-in and to determine the 95% probability density of ages for the nodes with a posterior probability of at least 0.1 in the tree. The trees were visualised and edited using FigTree 1.4.0 (http://beast.bio.ed.ac.uk/figtree).

The minimum spanning network of the ATPase 6 & 8 haplotypes was calculated using Arlequin 3.5.1.3. [61] with the TN93 model of sequence evolution, which was the best model available in Arlequin for this dataset. Visualization and editing were performed in Network 4.613 (http://www.fluxus-engineering.com/index.htm).

### Population genetic analyses

Descriptive statistics and genetic structure analyses were based on the ATPase 6 & 8 sequences only (with a few exceptions as mentioned below). Standard diversity indices, including number of haplotypes, number of variable sites, gene diversity and nucleotide diversity (π), were calculated in Arlequin 3.5.1.3.

Genetic differentiation between localities was measured by calculating pairwise-Φ_ST_ values in Arlequin 3.5.1.3. To calculate Φ_ST,_ we also used the TN93 correction, which was the best substitution model found by PartitionFinder 2 [51]. The statistical significance of Φ_ST_ was assessed using 10,000 permutations of individuals among the 19 localities. A Bonferroni correction was applied to adjust for the effect of multiple tests on the type I error. Because of the limited RAG1 dataset (S2 Data), we used this dataset to calculate the Φ_ST_ between pairs of areas (Upper Madeira, Lower Madeira/Central Amazon, and Negro and Tapajós rivers).

To assess the population structure without imposing *a priori* groupings of localities, we performed several spatial analyses of molecular variance (SAMOVA) using SAMOVA 1.0 [62], based on the ATPase 6 & 8 dataset. The SAMOVAs were performed on 1,000 simulated annealing steps. The significance of the fixation indices was tested by 10,000 permutations. We tested k-values (number of groups) ranging from 2 to 10, and considered the combination of high Φ_CT_ and small Φ_ST_ as an indicator of the best structure pattern. The geographical distance between localities in a riverscape is poorly estimated when based on the geographical coordinates of the localities (the distance separating two localities is generally not a straight line). Therefore, we used the first two axes of a Metric Multidimensional Scale (MDS) transformation performed on the geographical distance between pairs of localities, which was calculated by following the course of the rivers. Only the two first axes were used here because the analysis only allows two dimensions. The MDS analysis was performed using the package vegan 2.0–10 [63].

To test whether the different structuring processes envisaged for Amazonian fishes produce the expected pattern of genetic structuration in *T*. *albus*, we applied AMOVAs on the ATPase 6 & 8 dataset using Arlequin 3.5.1.3. We first tested for IBB by organizing the data into two groups according to their positions relative to the Teotônio Falls (the five sites from Upper Madeira upstream of the falls against the remaining sites). Then, we tested whether water colour was a driver of IBE by grouping together all blackwater/clearwater sites (three sites from Negro and Tapajós rivers and the Cautário River site, which is located upstream of the Teotônio Falls in a craton-originated river belonging to the Madeira River System) against whitewater sites (all remaining sites). Finally, we tested the two structuring factors together (IBB and water colour-driven IBE) by organizing the sites into three groups: *(i)* localities with blackwater/clearwater; *(ii)* localities in the Central Amazon and Lower Madeira downstream of the Teotônio Falls that display whitewater; and *(iii)* localities upstream of the Teotônio Falls in the Upper Madeira. As the Cautário River locality (cau) is located upstream of the Teotônio Falls but displays clearwaters, it can be placed either in group “*(i)*” or group “*(iii)*”. We took advantage of this situation to determine whether the waterfall or water colour structuring factor has a dominant role in explaining the genetic structure. When placing this locality in group “*(i)*”, a larger among-group variation would indicate that water colour has a stronger structuration effect than the Teotônio Falls; on the opposite, a larger among-group variation when placing this locality in group “(*iii*)” would indicate that the Teotônio Falls has a stronger structuration effect than water colour.

An additional AMOVA was also applied to the RAG1 sequences, even though the number of localities and the number of samples per locality were limited: aru (N = 5), cau (N = 4), ctl (N = 5), jac (N = 2), n1 (N = 4), pur (N = 2), sam (N = 4), slo (N = 3), sot (N = 4), and t1 (N = 4). The hypotheses of structuration by IBE and IBB were tested simultaneously, by testing both scenarios, i.e., *(i)* with the Cautário River grouped according to its water colour, and *(ii)* according to its geographical position (upstream of the Teotônio Falls), as presented in the previous AMOVA. Because of the presence of heterozygous sequences, we first run the coalescent-based Bayesian method implemented in PHASE 2.1 [64] to phase the diploid heterozygote sequences into haploid sequences. To confirm the robustness and consistence of PHASE results, we performed five independent runs with 10’000 iterations, considering recombination. The alleles showing the highest probability were kept.

### Redundancy analysis (RDA) for performing landscape genetics

To evaluate the contribution of the various population-structuring processes in explaining the genetic structure in *T*. *albus*, we applied a distance-based redundancy analysis (db-RDA). This method can be used when the response variable is a distance matrix (here the pairwise- Φ_ST_ between localities) and the explanatory variables are in the form of locality-wise values (vectors), either continuous or categorical. In our analysis, the only explanatory variable that was originally expressed as a distance matrix was the pairwise geographical distance; it was thus transformed into a vector format (S2 Appendix). The db-RDA method has the advantage of not being restricted to the use of Euclidean distances; any measure of similarity can be applied. Moreover, normality and homogeneity in the data are not mandatory, and the number of explanatory variables may be higher than the sampling size [65].

In our db-RDA, we set the explanatory variables as follows. *(I)* The *geographical distance* is represented by the vectors with positive eigenvalues of a Principal Coordinates of Neighbourhood Matrix (PCNM) [66], which was applied to the original geographical pairwise distance matrix between localities, with the distances calculated in kilometres following the course of the rivers. We kept only the vectors with positive eigenvalues because they describe the best the geographical distance between localities. *(II)* The *Teotônio Falls* is represented by a dummy variable, which is (0) upstream of the falls and (1) downstream of the falls. *(III)* The *water colour* (or water quality) is represented by the variables "water transparency" (in cm) and "water pH" (S6 Table), that were added to the model as independent variables. These variables are indicative of the different types of water colours in the Amazon because the whitewater rivers show neutral pH and low transparency (often only a few centimetres), whereas blackwater and clearwater rivers show pH values ranging from very acid to neutral and higher transparency (usually more than one metre) [67,68]. *(IV)* The *floodplain size* surrounding the sampling localities is represented by a size index that considers the area occupied by flooded zones during the rainy season for each site; this index varies from 0 to 1, with 0 representing the smallest floodplain size and 1 representing the largest floodplain size across our sampling localities. For details about how this index is calculated, see supporting information (S2 Appendix; S7 Table). *(V)* The *floodplain vegetation composition* was represented by the two first axes of a factor analysis performed on the relative abundance of each of the five following vegetation categories: *(i)* dense ombrophilous forest; *(ii)* open ombrophilous forest; *(iii)* tropical savannah or contact zones among savannahs, campinarana (dryland forest on white sand soil, slightly similar to a desert vegetation) and ombrophilous forest; *(iv)* secondary forest; and *(v)* anthropic areas influenced by agriculture or cattle raising. The vegetation categories and maps were obtained from the *Ministério do Meio Ambiente* of the Brazilian Government (S2 Appendix; S2 Fig; S8 Table; http://mapas.mma.gov.br).

The abovementioned variables were grouped into three classes according to their resulting pattern of isolation: IBD contains the variables representing geographical distance between localities *(i)*; IBB contains the variable associated with the landscape barrier, i.e., the Teotônio Falls *(ii)*; and IBE contains the variables associated with the water colour *(iii)*, the variables representing the floodplain size *(iv)* and the variables representing the floodplain vegetation composition (*v*).

As the variables show very different ranges, we first standardised them to have zero mean and variance equal one. To identify the variables that explain part of the genetic structure, we first ran a db-RDA on the full model (including all investigated variables) using the function “capscale” of the package vegan [63]. Then, we ran a db-RDA on nested models to identify the best model based on the Akaike information criterion (AIC). Because the db-RDA does not provide information on the relative contribution of each variable of the model, we performed a variance partitioning analysis using the function “varpart” of the package vegan [69] in the R environment [57]. The R script used to run these analyses is available in the Supporting Information (S1 Script).

## Results

### Genetic structure and demography

For the mtDNA fragment containing ATPase 6 & 8, 42 different haplotypes were identified (Table 1): nine were restricted to the whitewaters of the Upper Madeira (upstream of the Teotônio Falls), 19 were exclusively found in the whitewaters of the Lower Madeira/Central Amazon (downstream of the Teotônio Falls), and 10 were exclusive to the blackwaters/clearwaters of the Negro/Tapajós rivers. Two haplotypes were shared between the Lower Madeira/Central Amazon and the Negro/Tapajós, and one was shared between the Lower Madeira/Central Amazon and the Upper Madeira. A single haplotype was shared among the three areas. Concerning the nuclear RAG1 marker, 31 haplotypes (alleles) were recovered from the 37 diploid samples. Among them, 13 were found only in the Lower Madeira/Central Amazon, 10 were exclusive to the Negro/Tapajós rivers, and four were found only in the Upper Madeira. The Lower Madeira/Central Amazon shared one haplotype with the Negro/Tapajós, and three with the Upper Madeira. No haplotype was shared across the three areas.

The genetic structure assessed by locality pairwise-Φ_ST_ values based on the ATPase 6 & 8 indicated a strong genetic differentiation in *T*. *albus* among the three areas (S3 Fig). When the localities in the Upper Madeira were compared against those in the Lower Madeira/Central Amazon, the pairwise-Φ_ST_ values ranged from 0.17 (cau *vs* pur, according to locality names presented in Table 1) to 0.90 (sot *vs* aru). The highest Φ_ST_ values were found between the localities of the Upper Madeira and those of the Negro/Tapajós, and the values ranged from 0.71 (cau *vs* b1) to 0.90 (sot *vs* t1). The genetic differentiation between the pairs of localities of the Lower Madeira/Central Amazon and the Negro/Tapajós varied between 0.20 (a6 *vs* n1) and 0.81 (a5 *vs* t1). The majority of the pairwise comparisons involving localities from distinct areas were highly significant after Bonferroni correction. In contrast, pairwise-Φ_ST_ comparisons between localities from the same area were close to zero.

Using SAMOVA, we first asked for the best structure imposing two groups (k = 2). We found that the localities are grouped according to their water colour (Φ_CT_ = 0.53), irrespective of their position upstream or downstream of the Teotônio Falls barrier (Table 2). The only exception was one whitewater site (a6), located close to the mouth of the Tapajós, which clustered with blackwater/clearwater sites. When three groups were considered (k = 3), SAMOVA revealed a division of the localities according to their water colour and their position relative to the Teotônio Falls (Φ_CT_ = 0.70). We observed that the unique blackwater/clearwater locality upstream of the Teotônio Falls (Cautário River—cau) was grouped with the other localities upstream of the falls rather than with the localities sharing the same water colour. The Φ_CT_ values did not increase substantially with an increasing number of groups (k > 3), which suggests that an organization into three groups or populations best reflects the genetic structure of this species in the region considered.

**Table 2:** SAMOVA analyses based on the mitochondrial genes ATPase 6 & 8 of *Triportheus albus*. All fixation indices were significant at the 0.0001 level. First double bars in each line indicate the geographic position of the Teotônio Falls, whereas the second one represents the separation between whitewater and blackwater/clearwater rivers.

K	Fixation indices			Structure
	ΦSC	ΦST	ΦCT	
2	0.511	0.771	0.533	(cau sot ara slo jac **||** pur sam m1 a1 aru ctl a2 a3 a4 a5) (a6 **||** b1 n1 t1)
3	0.13	0.736	0.696	(cau sot ara slo jac) **||** (pur sam m1 a1 aru ctl a2 a3 a4 a5) (a6 **||** b1 n1 t1)
4	0.084	0.734	0.710	(cau sot ara slo jac) **||** (pur sam m1 a1 aru ctl a2 a3 a4) (a5) (a6 **||** b1 n1 t1)
5	0.062	0.732	0.714	(cau sot ara slo jac) **||** (pur sam m1 a1 aru ctl a2 a3 a4) (a5) (a6) **||** (b1 n1 t1)
6	0.041	0.728	0.717	(cau sot ara slo jac) **||** (pur sam m1 a1 aru ctl a2 a3 a4) (a5) (a6) **||** (n1) (b1 t1)
7	0.021	0.724	0.718	(cau sot ara slo jac) **||** (pur sam m1 a1 aru ctl a2 a3 a4) (a5) (a6) **||** (n1) (b1) (t1)
8	-0.005	0.720	0.721	(cau sot ara slo jac) **||** (pur) (sam m1 a1 aru ctl a2 a3 a4) (a5) (a6) **||** (n1) (b1) (t1)
9	-0.031	0.713	0.721	(cau sot ara slo jac) **||** (pur) (sam m1 aru ctl a2 a3 a4) (a1) (a5) (a6) **||** (n1) (b1) (t1)
10	-0.047	0.707	0.720	(cau) (sot ara slo jac) **||** (pur) (sam m1 aru ctl a2 a3 a4) (a1) (a5) (a6) **||** (n1) (b1) (t1)

To confirm the strong genetic structure based on the ATPase 6 & 8, we calculated pairwise-Φ_ST_ values between the three main groups using the nuclear RAG1 marker. Significant Φ_ST_ values (after Bonferroni correction) were found between the Upper Madeira and Negro/Tapajós (0.58; *P* < 0.00001), between the Lower Madeira/Central Amazon and Negro/Tapajós (0.55; *P* < 0.00001), and between the Upper Madeira and Lower Madeira/Central Amazon (0.12; *P* < 0.00001).

### Haplotype network, phylogeny and barcode divergence

**Fig 2:**
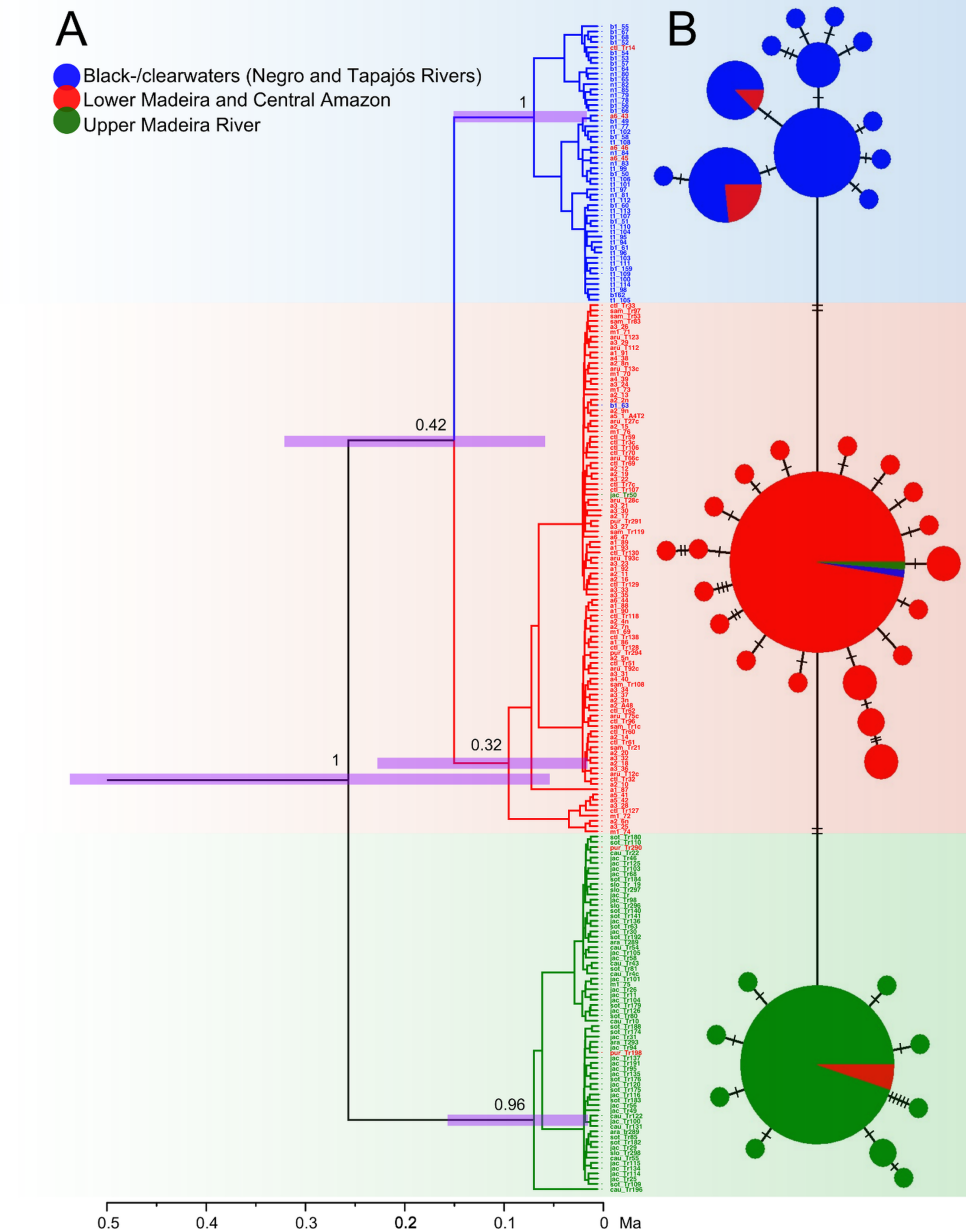
**A. Haplotype phylogenetic tree** using a coalescent tree prior, Bayesian Skyline, relaxed clock and mutation rate of 0.7% *per* Ma on the ATPase 6 & 8 sequences. Posterior probability values are indicated on the nodes. Pink bars correspond to the 95% highest posterior density (95%) for divergence times. **B. Minimum spanning network of ATPase 6 & 8 haplotypes from the three geographical regions.** In blue, localities in the Negro and Tapajós rivers; in red, Lower Madeira and Central Amazon; in green, the Upper Madeira. Hatch marks represent the number of mutations. The smallest and largest circles in the haplotype network represent 1 and 73 individuals, respectively.

The mtDNA (ATPase 6 & 8) haplotype network shows the presence of three haplogroups with few shared haplotypes among populations (Fig 2B). Haplotypes of the first haplogroup were mainly found in the Upper Madeira upstream of the Teotônio Falls, haplotypes of the second haplogroup were essentially found in the Lower Madeira/Central Amazon section, and haplotypes of the third haplogroup were mainly found in the Negro/Tapajós rivers. These haplogroups correspond well to the three populations revealed by SAMOVA (Table 1).

In the phylogenetic tree inferred using the Bayesian approach in BEAST2 (Fig 2A), we recovered three monophyletic lineages that correspond to the haplogroups described above. The first lineage grouped the individuals found in the Upper Madeira, and showed high posterior probability (PP = 0.99); the second lineage included the individuals from the Lower Madeira and Central Amazon (PP = 0.34); and the third lineage contained the individuals from the black- and clearwaters of the Negro and Tapajós rivers (PP = 0.95). The divergence between the blackwater/clearwater lineage and the whitewater lineage (Upper Madeira and Lower Madeira/Central Amazon) was estimated at 0.47 Ma (HPD 95% = 1.04–0.10 Ma). On the other hand, the age of separation of the Upper Madeira River population from the Lower Madeira/Central Amazon population was estimated at 0.24 Ma (HPD 95% = 0.49–0.07 Ma) (Fig 2A).

Considering the presence of three highly structured populations, we calculated the pairwise-divergence (K2P) between members of the different populations, using the barcode region of the COI gene. The highest pairwise genetic divergence was found between the Upper Madeira and Negro plus Tapajós lineages (0.7%), whereas the smallest divergence was between Upper Madeira and Lower Madeira/Central Amazon (0.1%). On the other hand, interspecific genetic divergence ranged from 5 to 18% (S2 Table).

### Testing explanatory factors with AMOVA

Using the ATPase 6 & 8 dataset, we applied four AMOVAs to test different scenarios underlying population structure (Table 3). When we tested only for IBB, grouping the localities according to their positions relative to the Teotônio Falls, we found a very high and significant Φ_CT_ (0.52). Then, we tested only for IBE (whitewater rivers *vs*. black- and clearwater rivers), resulting in a high and significant Φ_CT_ index (0.37; Table 3). We also tested simultaneously both patterns, IBB and IBE. As the Cautário River has clearwaters but is at the same time located upstream of the Teotônio Falls, we grouped it according to its water colour in the third AMOVA. We observed an increase in Φ_CT_ (0.55) when compared to the two previous AMOVA. However, when the Cautário River was grouped according to its position upstream of the Teotônio Falls (Upper Madeira group), the Φ_CT_ was higher than in all the other tests (0.69; Table 3). The AMOVA applied to the nuclear RAG1 sequences corroborated the results obtained with the mitochondrial ATPase 6 & 8 dataset, showing significant Φ_CT_ among the three groups (Table 4), especially when the Cautário River is grouped with the Upper Madeira population.

**Table 3:** Analysis of molecular variance (AMOVA) based on the mitochondrial genes ATPase 6 & 8. We tested for the structuration effect of (*I*) water colour (IBE), (*II*) Teotônio Falls (IBB), and (*III* and *IV*) the two structuration factors combined, and the Cautário River (cau) was tested as a member of the Upper Madeira group (*III*) or as a member of the blackwater/clearwater group (*IV*).

Structure	Source of variation	df	ss	vc	%	fi
I) IBE, whitewater *vs* blackwater/clearwater: (sot ara slo jac pur sam m1 a1 aru ctl a2 a3 a4 a5 a6) (cau b1 n1 t1)	among groups	1	63.3	0.61	37	0.37*
	among populations within groups	17	88.2	0.60	37	0.58**
	within populations	203	87.4	0.43	24	0.74**
II) IBB, Teotônio Falls: (cau sot ara slo jac) (pur sam m1 a1 aru ctl a2 a3 a4 a5 a6 b1 n1 t1)	among groups	1	98.4	0.97	53	0.53**
	among populations within groups	17	84.7	0.42	23	0.53**
	within populations	203	87.4	0.43	24	0.76**
III) Both IBE and IBB: (cau sot ara slo jac) (pur sam m1 a1 aru ctl a2 a3 a4 a5 a6) (b1 n1 t1)	among groups	2	162.5	1.13	69	0.69**
	among populations within groups	16	20.6	0.08	5	0.16**
	within populations	203	87.4	0.43	26	0.75**
IV) Both IBE and IBB: (sot ara slo jac) (pur sam m1 a1 aru ctl a2 a3 a4 a5 a6) (cau b1 n1 t1)	among groups	2	131.9	0.86	55	0.55**
	among populations within groups	16	51.3	0.27	17	0.38**
	within populations	203	87.4	0.43	28	0.72**

df = degree of freedom; ss = sum of squares; vc = variance components; % = percentage of variation; and fi = fixation indices. Locality codes are as in the Table 1. Significance after Bonferroni correction

*< 0.001

** < 0.0001.

**Table 4:** Analysis of molecular variance (AMOVA) based on the nuclear marker RAG1. We tested for the combined structuration effect of water colour (IBE) and Teotônio Falls (IBB). The Cautário River (cau) was tested as a member of the Upper Madeira group (*I*) or as a member of the blackwater/clearwater group (*II*).

Structure	Source of variation	df	ss	vc	%	fi
*I*) Both IBE and IBB: (cau sot slo jac) (pur sam aru ctl) (n1 t1)	among groups	2	64.2	1.23	44	0.44**
	among populations within groups	7	19.0	0.18	6	0.11*
	within populations	64	89.9	1.40	50	0.50**
*II*) Both IBE and IBB: (sot slo jac) (pur sam aru ctl) (cau n1 t1)	among groups	2	38.7	0.53	20	0.20
	among populations within groups	7	44.4	0.68	26	0.33**
	within populations	64	89.9	1.40	54	0.46**

df = degree of freedom; ss = sum of squares; vc = variance components; % = percentage of variation; and fi = fixation indices. Locality codes are as in the Table 1. Significance after Bonferroni correction

*< 0.05

**< 0.0001.

### Testing explanatory factors with db-RDA

To assess the association between the genetic structure and the variables that underlie the patterns of IBD, IBB, and IBE, we performed multiple regression analyses using the db-RDA method. Our response variable was the genetic structure expressed by the locality pairwise-Φ_ST_ matrix. The explanatory variables were *(i)* the geographical distance between localities as indicated by the first four axes of a PCNM on the geographical distance matrix (representing IBD); *(ii)* the position of the locality, i.e., upstream or downstream of the Teotônio Falls (representing IBB), as a categorical variable; *(iii)* the water colour indicated by the pH and the transparency of the water (representing IBE); *(iv*) the floodplain size (representing IBE) indicated by an index that expresses the maximal area surrounding the sampling locality that the main channel can inundate during rainy seasons; and *(v)* the floodplain vegetation composition (representing IBE) represented by the first two axes of a factor analysis of the composition of riparian vegetation (the two axes explaining 59% of the variation). The first vegetation axis was mainly explained by open vegetation (e.g., secondary vegetation, crops and pastures for cattle), whereas the second axis was explained by ombrophilous forest (S2 Appendix).

**Fig 3:**
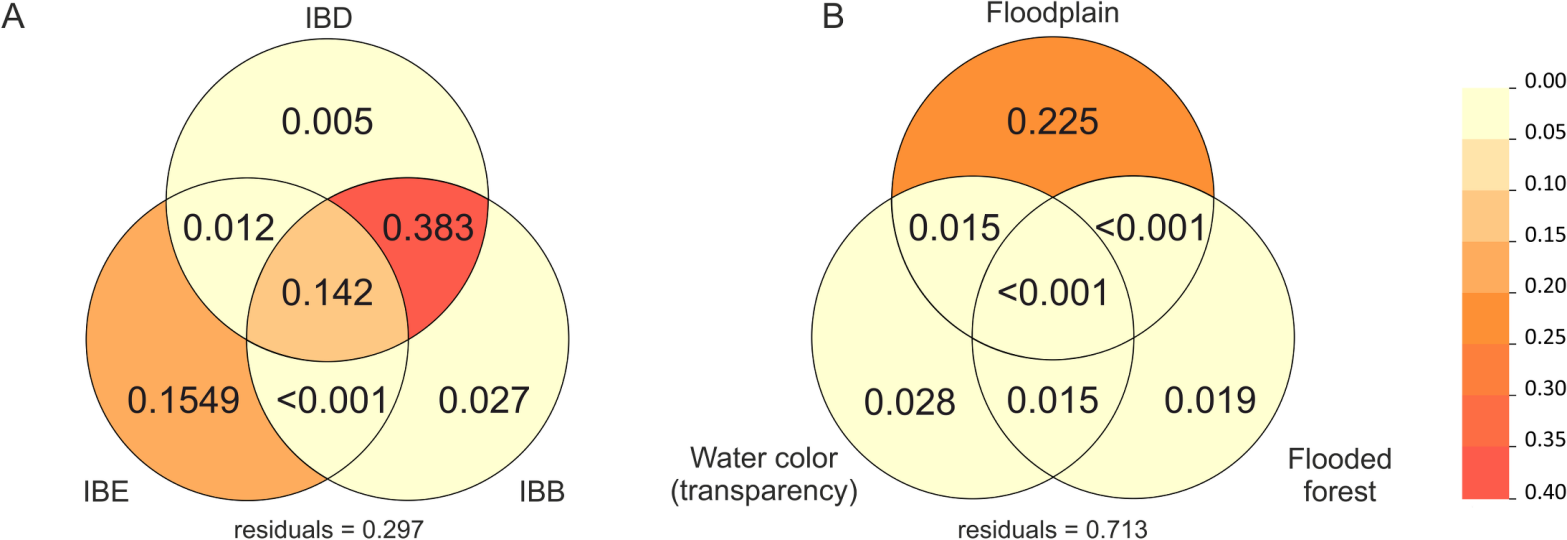
Variance partitioning analysis of the db-RDA results. **A.** The variation of the locality pairwise genetic differentiation (Φ_ST_) is explained by the variables underlying IBD (geographical distance), IBB (Teotônio Falls topographical barrier) and IBE (grouping three environmental characteristics: water colour/water transparency, floodplain size and flooded forest composition), and their interactions. The variance explained is indicated by the adjusted *R*^*2*^. **B**. Partitioning of the variance explained by the different variables underlying IBE: water colour/water transparency, floodplain size and flooded forest composition.

The optimal model for explaining the genetic structure was chosen according to its best AIC value (11.125); it was able to explain up to 70% of the genetic structure (*F* = 6.215; *P* = 0.001; Adj. *R*^*2*^ = 0.703) and contained six variables out of the initial 10: the geographical distance expressed as the first and fourth axes of the PCNM analysis (variables 1 and 2), location with regard to the waterfall (variable 3), the water transparency (variable 4), the floodplain size (variable 5), and the floodplain vegetation composition (first axis of the factor analysis; variable 6; S2 Appendix). The variance partitioning analysis was performed only on the variables that were included in the optimal model, and we grouped them according to the pattern of isolation they generate: IBD, IBB or IBE. The results showed that the variables driving IBE explained 15% of the variance of the genetic structure, whereas the variables underlying IBB and IBD (without their interactions) explained only 3% and 0.5%, respectively (Fig 3A). However, the interaction of the variables driving IBB and IBD (waterfall and geographical distance) explained up to 38% of the response variable, thus representing the largest contributor to the genetic structure in this species. We then performed a variance partitioning analysis on the three variables underlying IBE (Fig 3B). The results indicated that the floodplain size was the most important environmental variable driving the genetic structure and explained 23% of the variance, whereas the water transparency and vegetation composition explained only 3% and 2%, respectively.

## Discussion

To date, several processes of fish diversification in Neotropical rivers have been suggested, and most of them focus on historical events associated with neutral processes of speciation, such as events related to headwater captures, marine incursions, and uplifts of arches [70–76]. At the intraspecific level, less but significant work has been performed to understand how populations diversify through processes leading to IBD [8], IBB [30] or processes that are environmentally driven (IBE) [23]. Here, we examined the possibility that several of the proposed population diversification mechanisms can jointly determine population structure in an Amazonian fish species, and we assessed the relative role played by these processes.

To address these issues, we first inferred the population structure of the migratory fish *T*. *albus* in the central region of the Amazon Basin. Our results consistently revealed a very strong genetic structure with three main groups in the area considered. The first group inhabits the blackwater/clearwater of the Negro and Tapajós rivers, the second inhabits the whitewater of the Lower Madeira/Central Amazon, and the third inhabits the Upper Madeira, upstream of the Teotônio Falls.

The population genetic structure we uncovered in *T*. *albus* is so strong and remarkable that it raised the question whether they were in fact three populations or three species. Two different approaches were undertaken to clarify this issue. First, an ML tree based on the ATPase 6 & 8 including a subsample of *T*. *albus* and three other congeneric species supported the monophyly of *T*. *albus* (bootstrap value of 0.99). In addition, the intra-specific divergence (branch lengths) among *T*. *albus* lineages is substantially smaller than the inter-specific divergences (S3 Fig). Second, we sequenced and analysed the standard barcode marker, the mitochondrial COI gene, for a subsample of each *T*. *albus* population and other well-defined *Triportheus* species. The usual pairwise divergence (K2P) to delineate fish species based on this marker is 2% [48]. Here, the pairwise-divergence between members of the different *T*. *albus* populations ranged from 0.1 to 0.7% (S2 Table), while it was much higher among *Triportheus* species (5–18%). Therefore, the combination of the barcode approach with the classical ML tree reconstruction including congeneric outgroup species gives a clear indication that the three lineages we revealed in *T*. *albus* are intraspecific populations rather than different species.

### Isolation-by-distance

In nature, geographically distant populations commonly exchange fewer alleles than geographically close populations [6], leading to genetic differentiation related to geographical distance (IBD). The degree of differentiation can be determined by the dispersal capacity of the organism, which is often related to body size in fish [77]. Peterson and Denno's rule [78] states that IBD should be observed in species with an intermediate capacity of dispersal, whereas weak or no IBD pattern should be observed in species with a high capacity of dispersal and in species that show sedentary behaviour. This phenomenon can be explained on one side by the increased likelihood of strong dispersers to present high gene flow among populations, thereby limiting population structuration. On the other side, sedentary species are expected to show high population structure irrespective of the geographical distance separating populations.

Our db-RDA analysis showed a pattern of IBD in *T*. *albus* populations and, according to Peterson and Denno's rule, this would be indicative of an intermediate capacity of dispersal. This hypothesised migratory behaviour is supported by the observation that this species can perform medium-long distance migrations, of up to 100 km, mainly motivated by its reproductive behaviour [39]. At the beginning of the annual flood seasons, large schools undertake long downstream migrations along the tributaries to spawn in the main river channel. Eggs and alevins may drift further downstream along the main channel [13,79]. Although not well studied yet, juveniles are probably able to recolonize more upstream tributaries by active swimming over long distances [80], if major geographical breaks, such as rapids and waterfalls, are absent. This behaviour places *T*. *albus* in the list of intermediate dispersers at the Amazonian scale.

Peterson and Denno’s rule [78] seems to be valid in other Amazonian fishes. The giant catfishes of the genus *Brachyplatystoma*, for instance, can migrate more than 3,000 km to reproduce [39], and IBD has not been observed in species of this genus across the Amazon Basin [81]. In addition, the sedentary and widespread *Arapaima gigas* does not show an IBD pattern, even when the comparisons are performed at fine (< 25 km), meso (~100 km) or large scales (> 1,300 km) [82]. However, IBD is commonly detected in intermediate dispersers, such as *Serrasalmus rhombeus* [8] and *Prochilodus nigricans* [83], both showing migrations up to 100 km [39].

### Isolation-by-environment

In addition to geographical distance, other factors can drive population divergence in Amazonian fish populations. The characteristics of the environment and the diversity of habitats often modulate population connectivity by restraining gene flow between populations that inhabit different environments or are separated by unsuitable environments. This modulation occurs independently from the geographical distance separating the populations [84]. Such causes of gene-flow restriction between populations are referred to as IBE [85].

In our analyses, we found that the population structure of *T*. *albus* was apparently partly driven by water colour, which was represented by the "water transparency". Although many fish species are endemic to particular water colours in the Amazon Basin [18,19,86], a hypothetical adaptive explanation has been explicitly tested only recently, and the results indicated that many fish species or populations have adapted to different water qualities [22–25]. Consistent with our results, it was previously proposed that population divergence in *T*. *albus* has been triggered by adaptation to different water colours [22]. However, to prove an adaptive response of *T*. *albus* to the different Amazonian water colour, it would be necessary to conduct, in the future, transplant experiments in which individuals are translocated from their native water colour into another water colour, and their performance recorded. A fitness reduction triggered by the new environment would be an evidence for natural selection and adaptation to water colours.

Environmental differences other than water colour may trigger gene flow restriction among populations. Considering the ecological importance of the land-water ecotonal boundary, we hypothesised that differences in flooded environments could regulate gene flow among localities and contribute to the observed genetic structuration. We therefore tested the role of two other environmental factors in shaping population structure in *T*. *albus*: floodplain size and flooded riparian vegetation composition. The db-RDA results indicated that both variables contributed to the genetic structure. These variables were positively correlated with the pairwise locality genetic differentiation (Mantel *r* = 0.18 and 0.06, respectively), indicating more resistance to gene flow between localities with dissimilar floodplain size and dissimilar vegetation composition. Thus, we showed that floodplain heterogeneity in the Amazon Basin can drive fish population structuration in species that are dependent on these environments, and this factor might be even more important than water quality. As shown by our variance partitioning analysis, floodplain size was the habitat variable with the largest explanatory power.

Providing unambiguous evidence of the role of adaptation in structuration is challenging, especially in conditions where ex situ experiments are difficult to perform and time consuming. However, efficient and accessible genomic approaches are being developed to analyse genomes and provide evidence of adaptive genetic changes [87,88].

### Isolation-by-barrier

The stretch of rapids in the Madeira River that includes the Teotônio Falls has been shown to act as a barrier to gene flow at both the species and population levels for many aquatic and semi-aquatic vertebrates, such as dolphins [28,32,89], turtles [29], caimans [90], frogs [91], and fishes [30,31,83,92]. In this study, we showed that the Teotônio Falls are also an important contributor to the genetic structure in the migratory fish *T*. *albus*. This topographical barrier generated a strong pattern of IBB by dividing *T*. *albus* into two populations along the whitewater of the Madeira River. We estimated that the Upper Madeira and the Lower Madeira/Central Amazon populations diverged at approximately 0.24 Ma (HPD 95% = 0.49–0.07 Ma; Fig 2A). This estimate coincides with the relatively large time frame suggested for the origin of the Teotônio Falls, which is between 2.5 Ma [93] and 10,000 years [28].

Are the Teotônio Falls a stronger barrier to gene flow than local adaptation to different water colours? In our sample, a unique clearwater locality was observed upstream of the Teotônio Falls, the Cautário River, which is a tributary of the Guaporé River. According to the SAMOVA (Table 2), this locality groups with the sites located upstream of the Teotônio Falls rather than with the localities sharing the same water colour. Moreover, by performing AMOVAs, we obtained a higher genetic differentiation among populations when the Cautário locality is included in the Upper Madeira population than when it is included among the localities of black- and clearwater rivers (Tables 3 and 4). Further, the variance partition analysis indicated that the Teotônio Falls alone explained only 3% of the structuration pattern, whereas the combination of the Teotônio Falls with the geographical distance factor explained 38% of the structuration pattern. This indicates that waterfalls and long geographical distances limit the possibility of migration between localities with the same water colour, as it is the case for the Cautário locality that shares water colour with the Negro and Tapajós rivers. Additionally, we did not find evidence that the individuals from the Cautário River might form a subpopulation within the Upper Madeira’s population, indicating that divergence driven by water colour is not a rule and seems not to have occurred independently many times in the evolution of *T*. *albus*.

### Alternative historical hypothesis

The dynamic and complex history of the Amazon watershed should be scrutinised to determine whether modern changes have masked ancient configurations responsible for the currently observed patterns. Here, we discuss alternative historic processes that might explain the origin of the three *T*. *albus* populations.

The divergence of the population inhabiting whitewater rivers from the population of black-and clearwater rivers could have been triggered by a potential partial isolation of the Upper Amazon Basin from the Lower Amazon Basin during the cold and dry periods of the Pleistocene. The hypothesis we propose here relies on the possibility that during the glacial periods, the water discharge of the numerous Andean tributaries of the Upper Amazon River, carrying whitewaters, was drastically reduced due to decreased precipitation and the lack of Andean ice sheet melting [94]. Five of the last major glacial periods [95] occurred in the same time window than the estimated age of the divergence between the whitewater population and the black- and clearwater population (HPD 95% = 1.04–0.10 Ma; Fig 2A). It is likely that the reduced water flow of the Upper Amazon was retained by topographical reliefs, such as the Purus Arch, a low-ridge mountain range located immediately upstream of the meeting of the Amazon and Negro rivers. The Purus Arch is suggested to have been the border between the eastern and the western Amazonian drainages during the Miocene, prior to the current configuration of the Amazon River [70], and has been suggested to be a likely reason of population structuration in some cichlid species [9,21]. Thus, the Purus Arch might also have acted as a topographical barrier during the major glacial periods of the Pleistocene, driving to population divergence between the Upper Amazon Basin, containing whitewater rivers, and the Lower Amazon Basin, composed of black-and clearwater rivers. Although a whitewater outlet of the Upper Amazon could have flowed into the Lower Amazon, this whitewater contribution would have been highly diluted by the predominant black- and clearwaters coming from the main tributaries of the Lower Amazon, such as the Negro and the Tapajós rivers. Consequently, the main course of the Lower Amazon river would have carried black- or clearwaters. Since the water quality is expected to have been different in both sides of the Purus Arch, the divergence between the whitewater population and the black- and clearwater population was not only driven by this topographical barrier, but also reinforced by ecological adaptation to the different water colours. This adaptation would explain why both populations have not admixed after the last glacial period, when they experienced a secondary contact. Our hypothesis can also explain why *T*. *albus* specimens from the Negro and the Tapajós rivers (black- and clearwater rivers), although separated by the whitewaters of the Lower Amazon, still share the same mitochondrial haplotypes today. This would be due to the fact that during the glacial periods, the Lower Amazon was composed of black- or clearwaters, generating a continuous habitat with homogenous water quality linking all the tributaries of the Lower Amazon Basin. Accordingly, it is only during the interglacial periods that the lower course of the Amazon was dominated by the Andean whitewaters, evidencing that the contemporary environmental condition does not correspond to the historical condition under which the divergence occurred. It is notable that a similar pattern of population divergence found in other species [23,24] could be explained by this hypothesis.

Concerning the isolation of the *T*. *albus* population inhabiting the Upper Madeira from the Lower Madeira/Central Amazon, the Pleistocene climate fluctuations might also have played a role. The reduced water flow in certain river sections might have led to the formation of discontinuous reservoirs located in more humid areas (decreasing the size of the hydroshed basins), thus creating fragmented aquatic refuges (Haffer’s refuge hypothesis [96]). The fragmentation of the habitat may have caused fish diversification at the population level [8,81,97,98]. The likely presence of a refuge in the Upper Madeira section formed during the last Pleistocene glaciation period [99] has been proposed as the mechanism underlying the population structuration in *Serrasalmus rhombeus* [8] and *Cichla pleiozona* [98], and this mechanism may provide an alternative explanation for the origin of the Upper Madeira population of *T*. *albus*.

Another possible origin for the Upper Madeira population is based on the recent reconstruction of the Amazon paleodrainage pattern. During the Pleistocene-Holocene, the current Upper-Middle Madeira River was probably not connected to the Lower Madeira but rather was a tributary of the Purus River, another major affluent of the Amazon located westward [100,101]. According to this drainage capture reconstruction, the Upper Madeira population could have diverged from the current Lower Madeira/Central Amazon population because of this historical separation. This possible event has already been suggested to explain genetic similarity, higher than expected, between populations of *Prochilodus nigricans* from the Upper Madeira and Upper Purus [83] as well as between populations of the peacock cichlids of the genus *Cichla* [102]. This hypothesis could be tested in future works by collecting *T*. *albus* specimens from the Upper and Middle Purus River, and assessing whether they are more closely related to the Upper Madeira population than to other populations.

Nevertheless, the alternative hypotheses explaining the origin of some *T*. *albus* populations do not challenge our results and interpretation. First, the alternative scenarios to explain the isolation of the Upper Madeira population (Pleistocene refuge or drainage capture) include a geographical division that coincide with the Teotônio Falls and the rapids of the Madeira River. Both hypotheses involve physical barriers to fish dispersal and gene flow that occurred either via river sections with highly reduced flow during the dry periods of the late Pleistocene or the physical discontinuity between the Upper-Middle Madeira River (flowing into the Purus) and its lower section. On the other hand, our alternative explanation for the divergence between the whitewater population and the black- and clearwater population would include two processes acting concomitantly, the partial barrier represented by the Purus Arch (a neutral process) and the adaptive response to different water colours. Therefore, our results and interpretations of the processes leading to IBD, IBE and IBB in shaping the genetic structure of the Amazonian *T*. *albus* appear to hold true regardless of the hypothesised mechanisms underlying their origin.

Even though historical processes could have been the main evolutionary forces driving the intraspecific diversification in *T*. *albus*, it is still possible that the contemporary processes tested here are acting in maintaining the genetic structuration. Nonetheless, further research is needed to disentangle this challenging issue.

### Conclusions and conservation considerations

Our results show that the process of fish population diversification in the Amazon is complex and not limited to a single mechanism, which has been the focus of most studies. Here, we used a general method to demonstrate for the first time that interactions among several processes have had an impact on the structuration of the populations of an Amazonian fish. These mechanisms include geographical distance that lead to IBD, physical barriers that lead to IBB, and likely adaptation to environmental conditions related to differences in water colour, floodplain size and floodplain vegetation composition that lead to IBE. Moreover, the analyses of variance partitioning allowed us to unravel the relative role of these variables and the importance of their interactions. Hence, several processes have driven the population structuration in *T*. *albus*. Our results exemplify the power of the methodologies applied here, which can easily be implemented for any species.

The eventful history of the Amazon Basin, the varied environments, the richness of the communities and the synergistic action of several population diversification processes may accelerate population divergence to the point of speciation, and could partly account for the high speciation rate characterizing this basin. Although the relationship between levels of intraspecific structuration and the number and age of species within a lineage has been poorly investigated, a clear correlation between levels of within-species genetic structure and speciation rates has been shown for oceanic islands [103]. Similarly, the tremendous Amazonian fish species richness might be explained by a higher speciation rate triggered by the cumulative action of many population structuration processes.

In the Amazon Basin, the major biodiversity conservation efforts have been centred on the species level or higher taxonomic levels, and they often neglect intraspecific diversity. Thus, opportunities to preserve the inherent short-term evolutionary processes acting at the intraspecific level are often missed [104]. We argue that the multiple population structuration processes and the population diversity generated by these processes should be preserved to maintain the variety of evolutionary paths and allow for the emergence of new diversity. Such preservation is especially important because human modifications of the Amazon landscape are rapidly altering the structuration processes and threatening population diversity.

The recent construction of two dams along the Madeira River remodelled the landscape in the stretch of rapids containing the Teotônio Falls and has permanently flooded a vast upstream area, which has resulted in a complete disruption of the floodplain dynamics. The dams also artificially modulate the downstream flow, which affects the dynamics of the downstream floodplains. The dams cause obvious disturbances, and they have likely taken on the role of barrier to gene flow previously attributed to the Teotônio Falls. However, the dams do not fully mimic the functions of the waterfalls on the biology of local fishes because certain migratory species were able to cross the falls and others experienced only marginal disruptions to their dispersal [30]. Fish ladders have been constructed in the dam located more downstream and closest to the former Teotônio Falls, to facilitate the movements of fish species. However, if the ladders are not selective enough, they may facilitate the passage of fish species that naturally would not cross the falls, such as *T*. *albus*, disrupting the barrier effect and leading to the genetic homogenization of upstream and downstream populations. Monitoring fish transit through the ladders would certainly provide key information that could be used to better estimate the consequences of the dams on the genetics of populations.

The imminent construction of dams in other main Amazonian tributaries, such as the Negro, Tapajós and Xingu rivers, represents another threat. These rivers are home to ancient rapids and waterfalls and convey blackwater and clearwater, which are known to trigger fish diversification. To develop conservation strategies in these areas, evaluations must be performed to determine the species diversity and the role played by landscape peculiarities, such as waterfalls, water colour and floodplain characteristics, which are drastically changed by the construction of dams. As demonstrated in the present work, these features are important drivers of the genetic differentiation in fishes across the Amazon Basin.

## Supporting information

S1 Appendix Amplification and sequencing. (PDF)

S2 Appendix Distance-based Redundancy Analysis (db-RDA). (PDF)

S1 Table Primers for amplification and sequencing mitochondrial and nuclear genes in *Triportheus albus*. ATPase 6 & 8 primers were proposed by Berminghan & Martin (1998), whereas the primers to amplify RAG1 and COI were developed in the present study.
(PDF)

S2 Table Estimates of COI sequence divergence between pairs of *Triportheus* species, and between pairs of lineages of *T*. *albus*. BC (population from the Negro and Tapajós rivers, black- and clearwater rivers); CA (population from the Central Amazon and Lower Madeira River, whitewater rivers); and UM (population from the Upper Madeira, whitewater river). The analysis was based on the barcode region (647 bp) and was conducted in Mega v. 6.06 using the Kimura 2-parameters model (K2P).
(PDF)

S3 Table Haplotypes of ATPase 6&8. (PDF)

S4 Table Haplotypes of RAG1. (PDF)

S5 Table Haplotypes of COI. (PDF)

S6 Table Average values of pH and transparency of the water in different sites across the Amazon Basin. (PDF)

S7 Table Floodplain index. Total Area of the polygon (a), area of the main channel (b) and the area occupied by lateral lakes and water bodies (c). The index was calculated using this formula: [(*c*/*a*) + (*c*/(*b*+*c*)) + (*c*/*b*)]/3.
(PDF)

S8 Table Area (in km^2^) occupied by each type of vegetation around the sampling localities. Secondary forest (SF); anthropic area (AP); open areas, including savannahs, forested campinaranas, and contact zone area between savannahs and ombrophilous forest (SCO); dense ombrophilous forest (DO); and open ombrophilous forest (OO). Original data comes from the *Ministério do Meio Ambiente* of the Brazilian Government (http://mapas.mma.gov.br/mapas/aplic/probio/datadownload.htm).
(PDF)

S1 Fig **A. Maximum likelihood tree of *Triportheus albus* and close species.** The tree shown here is based on the ATPase 6 & 8 marker. It contains a subsample of *T*. *albus* representatives of each of the three lineages: (i) in green, samples from the Upper Madeira lineage; (ii) in red, samples from the Lower Madeira and Central Amazon lineage; and (iii) in blue, samples from the black- and clearwaters of the Negro and Tapajós rivers. Representatives of three other congeneric species are included. This tree supports the monophyly of *T*. *albus*. Values in the nodes represent node support, which were estimated by 1000 bootstrap replicates. Values lower than 50 are omitted. **B.** As the *T*. *albus* lineages show particularly very short branch lengths, we present the same tree transformed into a cladogram.
(TIFF)

S2 Fig Vegetation map of the studied area. The polygons represent the area in which the proportion of the different vegetation categories was calculated for each sampling site. This map and those with higher resolution (1:250’000), which were used for a more precise estimation of the proportion of each vegetation categories, are provided by the Brazilian Government (*Ministério do Meio Ambiente*) and are freely available in the public domain (http://mapas.mma.gov.br/mapas/aplic/probio/datadownload.htm). Vegetation categories present in the original maps but which were not found in the areas analysed in this study were omitted in this map.
(TIF)

S3 Fig Localities pairwise Φ_ST_ matrix based on ATPase 6 & 8. Colours intensity represents the significance level.
(TIFF)

S1 Data The *ATPase synthase subunit six and subunit eight* (ATPase 6 & 8) alignment of *Triportheus albus* and outgroups. (FASTA)

S2 Data The recombination activating protein 1 (RAG1) alignment of *Triportheus albus*. (FASTA)

S3 Data The *cytochrome c oxidase subunit I* (COI) alignment of *Triportheus albus* and other *Triportheus* species. (FAS)

S4 Data File containing the information about the priors and parameters used in BEAST2. This XML file was used to obtain the phylogenetic reconstruction and age estimates of the lineages of *Triportheus albus*, presented in Fig 2 of the main text.
(XML)

S1 Script R Script used to run the distance-based Redundancy Analysis (db-RDA). (R)
