# Supplementary material for: Multifactorial genetic divergence processes drive the onset of speciation in an Amazonian fish
Source: PLoS One. 2017 Dec 20;12(12):e0189349. doi: 10.1371/journal.pone.0189349 (PMC5738069; doi:10.1371/journal.pone.0189349)
Supplement: S1 Appendix — (PDF) [file pone.0189349.s001.pdf]

Supporting information 1 (S1)

Amplification and sequencing

The mitochondrial *ATPase synthase subunits six and eight* (ATPase 6 & 8) genes and the *cytochrome C oxidase subunit I* (COI) gene, and the nuclear gene encoding the *recombination activating protein 1* (RAG1) were amplified using the primers described in Table S1. PCR reactions were run in 50 µl reactions, consisting in 5 µl 10× reaction buffer, 1 µl dNTP mix at 10 mM each, 1 µl of each primer at 10 µM, 1 µl $MgCl_2$ at 25 mM, 0.25 µl *Taq* DNA polymerase equivalent to 1 U of polymerase per reaction, 1–10 µl DNA (depending on the DNA concentration). PCR profiles were as follows:

ATPase 6 & 8:
(i) 2 min at 94 ºC (initial denaturation);
(ii) 30 s at 94 ºC;
(iii) 30 s at 53 ºC;
(iv) 60 s at 72 ºC;
(v) 5 min at 72 ºC (final elongation).
Steps ii–iv were repeated 40 times.

COI:
(i) 2 min at 94 ºC (initial denaturation);
(ii) 30 s at 94 ºC;
(iii) 30 s at 52 ºC;
(iv) 60 s at 72 ºC;
(v) 5 min at 72 ºC (final elongation).
Steps ii–iv were repeated 38–40 times.

RAG1:
(i) 2 min at 94 ºC (initial denaturation);
(ii) 30 s at 94 ºC;
(iii) 30 s at 57 ºC;
(iv) 100 s at 72 ºC;
(v) 5 min at 72 ºC (final elongation).

Steps ii–iv were repeated 38–40 times.

The PCR products were checked on agarose gels and sent for sequencing at Macrogen Inc. (Amsterdam, Netherlands). Accession numbers of the haplotypes sequences can be found in the Tables S3–S5. Complete alignments are available at S1–S3 Data.
