# Supplementary material for: Multifactorial genetic divergence processes drive the onset of speciation in an Amazonian fish
Source: PLoS One. 2017 Dec 20;12(12):e0189349. doi: 10.1371/journal.pone.0189349 (PMC5738069; doi:10.1371/journal.pone.0189349)
Supplement: S2 Appendix — (PDF) [file pone.0189349.s002.pdf]

Supporting information 2 (S2)

Distance-based Redundancy Analysis – db-RDA

We performed distance-based redundancy analysis (db-RDA) to unravel the variables explaining the genetic differentiation of *T. albus* among localities. To do so, we first calculated the genetic distance between each pair of localities (the pairwise-Φ_{ST} distance matrix). Φ_{ST} values were computed in Arlequin 3.5.1.3 [1], with TN93 correction. Then we used the pairwise-Φ_{ST} matrix as the response variable in the db-RDA. Some of the environmental data that were used as explanatory variables were pre-processed in order to reduce dimensionalities and to fit them in the db-RDA models, as described below.

<u>Explanatory variables</u>

i) Geographical distance

The geographical distance between each pair of sampling site was calculated by considering the course of the rivers, instead of using a straight-line Euclidean distance extracted from the geographical coordinates. To transform the matrix of geographical distance into vectors, we applied a Principal Coordinates of Neighbour Matrices (PCNM) by using the package PCNM [2] and its function "PCNM", following the methodology described in [3]. This is a method of decomposition of the spatial relationships among sites in which vectors with positive eigenvalues express very well the distribution of the sampling sites in the geographical space. It has been confirmed the spatial structure reconstructions by using thFis method, and showed that PCNM has still another advantage, as, in many cases, it is very powerful even for small data series [3]. In our analyses, the first four axes (out of a total of 10) were found to have positive eigenvalues and were kept for the db-RDA. These axes were respectively called "geo1", "geo2", "geo3" and "geo4".

ii) Teotônio Falls

The Madeira River is characterized by a long stretch of several waterfalls and rapids. The major waterfall, the Teotônio Falls, has been found to play a strong role in the distribution of the aquatic fauna [4]. To test if this waterfall can be the underlying cause of a pattern of IBB in *Triportheus albus* populations, we incorporated a dummy variable in the model, according to the position of each sampling site relative to the Teotônio Falls: the sites were categorized either as "0" if located upstream of the Teotônio Falls (cau, sot, ara, slo and

jac), or “1” if located downstream of the falls (pur, sam, m1, ctl, aru, a1, a2, a3, a4, a5, a6, b1, n1, t1). For the sampling site abbreviations, see Table 1 in the main article.

iii) Water colour

To analyse the contribution of the different water colours (white-, clear- and blackwaters) in leading to an IBE pattern, we used the two most conspicuous variables characterizing these water colours in the Amazon Basin: water transparency and pH (S6 Table). Many Amazonian rivers, including the Amazon River itself, have their headwaters located in the Andean Mountains (e.g. Madeira, Japurá and Purus Rivers). They are commonly referred to as *whitewater rivers* because of the elevated concentration of suspended sediments in their whitish muddy waters. This leads to a very low transparency of the water (often a few centimetres only). The high amount of sediment (~300 tons/km^2 year; [5] controls directly the pH of the water, which varies from slightly acid to neutral (pH 6–7) [6].

On the contrary, *blackwater rivers*, as the Negro River, drain very ancient terrains, and carry a low amount of suspended sediment (~11.5 tons/km^2 year; [5]. Thus, blackwaters are transparent (often more than 1 m of underwater visibility). Moreover, the high amount of humic and fulvic acids in the these rivers is directly linked to their high acidity (pH 4–5; [6] and reddish-brown to black colour.

Likewise, *clearwaters rivers* also originate in the Precambrian areas of South America, and show high water transparency, as the sediment loaded in their waters is also very small (~15 tons/km^2 year; [5]). The colour of clearwater rivers is greenish. The pH of clearwaters is more variable, ranging from very acid to alkaline [7]. In the Amazon Basin, Tapajós and Xingu Rivers are the most representatives among clearwater rivers.

In our study area, one sampling site was located in the blackwaters of the Negro River (n1), three in clearwater rivers (the Branco River, b1, belonging to the Negro River Basin; the Tapajós River, t1; the Guaporé River, cau, belonging to the Madeira River Basin), while all the remaining sites were located in the whitewaters of the Amazon and Madeira Rivers.

iv) Floodplain index

An important fraction of the Amazonian fish fauna, including the species *Triportheus albus*, is very dependent on the flood pulse and floodplains for growth, shelter and feed activities and reproduction [8]. Since the extension of the floodplain is variable in size along the studied area, we predicted that the genetic structuration in the Amazonian fishes might be

explained partially by this environmental characteristic. For instance, in the Guaporé–Mamoré river stretch, the floodplain is relatively larger, and a substantial area of the riparian forest is inundated periodically as well as in the Lower Madeira and Central Amazon [9]. Yet, in stretches were the Madeira River is more entrenched, for instance downstream of the Guaporé–Mamoré area, floodplains are almost absent or narrow [4,10]. Hence, we included as explanatory variable in the db-RDA a floodplain index that depicts the prominence (size) of the floodplain for each sampling site (S7 Table). Our aim was to assess whether the floodplain size was a contributor to the genetic structuration of a fish species that is dependent on this environment.

The floodplain index was calculated by using satellite images available in Google Earth's 7.1.2.2041, in which we drew polygons around each sampling site. The size of the polygons was proportional to the width of the main channel. To determine the extension of the polygon in each side of the main channel, we multiplied the main channel width by three; the length, however, was defined by multiplying the main channel width by 10. In this way, we were able to encompass the marginal forest potentially inundated, and the main channel of the river in the centre. Then we calculated in km^2 (a) the polygon area, (b) the area occupied by the main channel in the polygon during dry seasons, and (c) the area occupied only by the lateral lakes and other water bodies during the rainy season. Based on these values, the floodplain index was calculated by combining the ratios: $[(c/a) + (c/(b+c)) + (c/b)]:3$. For one particular sampling sites, the Ariaú Channel, this calculation was not possible because it is located in the floodplain of the Amazon River and not on the main stream. We thus used the value of the closest locality along the main course of the Amazon River (a1).

v) Flooded vegetation composition,

The inundated forest is also intrinsically associated to the life cycle of a large fraction of Amazonian fish species [11]. Being omnivorous, *Triportheus* species feed predominantly on seeds, fruits and terrestrial insects during the flood season [12–14]. Thus, the vegetation composition of the flooded forest might also determine the quality of the environment and may explain patterns of distribution and structuration. To test the prediction that spatial variation in the flooded forest composition can lead to IBE, we determined the vegetation composition of the flooded areas surrounding the collection localities. We used high-resolution vegetation maps (1:250'000) freely available at the *Ministério do Meio Ambiente* of the Brazilian Government (S1 Fig; http://mapas.mma.gov.br/mapas/aplic/probio/datadownload.htm). We drew new polygons

around each sampling site following the same protocol used to calculate the floodplain index. The software ImageJ version 1.48 was used to visualize the images and to determine manually the area (km^2) occupied by each vegetation type in the polygons (S1 Fig). A total of 17 categories of vegetation were arranged in five groups: *I)* Dense Ombrophilous forest (DO), a typical Amazonian forest with elevated trees and a canopy at 50 m high; *II*) Open Ombrophilous forest (OO), characterized by its reduced forest density and present in areas of transitions between the typical Amazonian forest (DO) and other vegetation categories; *III*) Areas of tropical savannah or contact zone areas between Savannahs, Campinarana and Ombrophilous forest (SCO), which include more open areas composed of smaller trees (stems with approximately 0.5 m high). The two remaining areas are places modified by human activities, including: *IV*) secondary forest (SF) and *V*) anthropic areas influenced by agriculture and/or cattle raising (AP). For each locality polygon, the area of each of these five vegetation groups was estimated (S8 Table).

We performed a factor analysis on the raw data of the area (in km^2) occupied by each type of vegetation around the sampling sites, using the function "factanal" of the package Vegan 2.0-10 [15]. The first two dimensions retrieved 59% of the variation of the data and were retained in the db-RDA models. They were called "veg1" and "veg2". The first dimension, veg1 (37.5% of the variation), was mainly explained by SF, SCO and AP, which presented loadings of 0.87, 0.72 and 0.67, respectively. The second axis, veg2 (21.2% of the variation) was essentially led by DO (0.99).

<u>db-RDA models</u>

We first ran a db-RDA on the full model, i.e. including all the explanatory variables that might explain genetic structuration and lead to patterns of isolation (IBD, IBB and IBE). This analysis was performed using the function "capscale" of the package Vegan [15], in the R environment [16]. Since each explanatory variable showed a different range of variation, they were standardized *a priori* to mean equal zero and standard deviation equals 1, using the function "scale". Subsequently, db-RDA was run on nested models to identify the best model taking into consideration the Akaike information criterion (AIC). We also assessed the multicollinearity among the predictor variables by calculating the variance inflation factor (VIF). The VIF was smaller than 8 for our best model, indicating no multicollinearity in the variables [17].

After identifying the best model, we aimed to quantify the contribution of each variable in explaining the genetic structuration. To do so, we used the function "varpart" of

the package Vegan, and calculated the percentage (measured as adjusted-R^2), of explained variance obtained by the db-RDA [18]. Since this function limits the analysis to four variables (or set of variables), we performed this analysis in two stages. First we assessed the contribution of the variables grouping them according to their isolation pattern: IBD was composed of the variable geo1 + geo4; IBB was composed of the variable waterfall; IBE was composed of the variables water transparency + floodplain size + vegetation composition. Then, we performed the same analysis but evaluating the contribution of the variables that might lead to IBE only (water transparency, floodplain size, vegetation composition).

The script we used to run these analyses with all the steps is available at http://genev.unige.ch/research/laboratory/Juan-Montoya.

References

1. Excoffier L, Laval G, Schneider S. Arlequin (version 3.0): An integrated software package for population genetics data analysis. Evol Bioinforma Online. 2007;1: 47–50.

2. Legendre P, Borcard D, Blanchet FG, Dray S. PCNM: MEM spatial eigenfunction and principal coordinate analyses. R Package Version 21-2r109 2013. 2013;

3. Borcard D, Legendre P. All-scale spatial analysis of ecological data by means of principal coordinates of neighbour matrices. Ecol Model. 2002;153: 51–68. doi:10.1016/S0304-3800(01)00501-4

4. Torrente-Vilara G, Zuanon J, Leprieur F, Oberdorff T, Tedesco PA. Effects of natural rapids and waterfalls on fish assemblage structure in the Madeira River (Amazon Basin). Ecol Freshw Fish. 2011;20: 588–597. doi:10.1111/j.1600-0633.2011.00508.x

5. Latrubesse EM, Stevaux JC, Sinha R. Tropical rivers. Geomorphology. 2005;70: 187–206. doi:10.1016/j.geomorph.2005.02.005

6. Junk WJ, Wittmann F, Schöngart J, Piedade MTF. A classification of the major habitats of Amazonian black-water river floodplains and a comparison with their white-water counterparts. Wetl Ecol Manag. 2015;23: 677–693. doi:10.1007/s11273-015-9412-8

7. Sioli H. The Amazon: limnology and landscape ecology of a mighty tropical river and its basin. Dordrecht [Netherlands]; Boston; Hingham, MA, USA: W. Junk ; Distributors for the U.S. and Canada, Kluwer Academic Publishers; 1984.

8. Saint-Paul U, Zuanon J, Correa MAV, García M, Fabré NN, Berger U, et al. Fish Communities in Central Amazonian White- and Blackwater Floodplains. Environ Biol Fishes. 2000;57: 235–250. doi:10.1023/A:1007699130333

9. Irion G, Junk WJ, Mello JASN de. The Large Central Amazonian River Floodplains Near Manaus: Geological, Climatological, Hydrological and Geomorphological Aspects. In: Junk DWJ, editor. The Central Amazon Floodplain. Springer Berlin Heidelberg; 1997. pp. 23–46. Available: http://link.springer.com/chapter/10.1007/978-3-662-03416-3_2

10. Torrente-Vilara G, Zuanon, J, Amadio S, Doria CRC. Biological and ecological characteristics of Roestes molossus (Teleostei: Cynodontidae), a night hunting characiform fish from upper Madeira River, Brazil. Ichthyol Explor Freshw. 2008;19: 103–110.

11. Goulding M. The Fishes and the Forest: Explorations in Amazonian Natural History. Berkeley, Los Angeles and London: University of California Press; 1980.

12. Yamamoto KC, Soares MGM, Freitas CE de C. Feeding of Triportheus angulatus (Spix & Agassiz, 1829) in the Camaleão lake, Manaus, Amazonas state, Brazil. Acta Amaz. 2004;34: 653–659. doi:10.1590/S0044-59672004000400017

13. Maia LA, Santos LM dos, Parolin P. Seed germination of Bothriospora corymbosa (Rubiaceae) recouped of digestive tract of Triportheus angulatus (sardine) in Camaleão Lake, Central Amazonian. Acta Amaz. 2007;37: 321–326. doi:10.1590/S0044-59672007000300002

14. Pereira J de O, Silva MT da, Vieira LJS, Fugi R. Effects of flood regime on the diet of Triportheus curtus (Garman, 1890) in an Amazonian floodplain lake. Neotropical Ichthyol. 2011;9: 623–628. doi:10.1590/S1679-62252011005000029

15. Oksanen J, Blanchet FG, Kindt R, Pierre Legendre, Minchin PR, O'Hara RB, et al. vegan: Community Ecology Package [Internet]. 2014. Available: http://cran.r-project.org/web/packages/vegan/index.html

16. R Development Core Team. R: A language and environment for statistical computing. In: R: A language and environment for statistical computing [Internet]. 2008 [cited 1 Jan 2017]. Available: http://www.R-project.org.

17. Borcard D, Gillet F, Legendre P. Numerical Ecology with R [Internet]. New York, NY: Springer New York; 2011. Available: http://link.springer.com/10.1007/978-1-4419-7976-6

18. Peres-Neto PR, Legendre P, Dray S, Borcard D. Variation partitioning of species data matrices: estimation and comparison of fractions. Ecology. 2006;87: 2614–2625. doi:10.1890/0012-9658(2006)87[2614:VPOSDM]2.0.CO;2
