# Supplementary material for: Multifactorial genetic divergence processes drive the onset of speciation in an Amazonian fish
Source: PLoS One. 2017 Dec 20;12(12):e0189349. doi: 10.1371/journal.pone.0189349 (PMC5738069; doi:10.1371/journal.pone.0189349)
Supplement: S1 Table — ATPase 6 & 8 primers were proposed by Berminghan & Martin (1998), whereas the primers to amplify RAG1 and COI were developed in the present study. (PDF) [file pone.0189349.s003.pdf]

S1 Table. Primers for amplification and sequencing mitochondrial and nuclear genes in *Triportheus albus*. ATPase 6 & 8 primers were proposed by Berminghan & Martin (1998), whereas the primers to amplify RAG1 and COI were developed in the present study.

Gene	Step	Primers	Sequence
ATPase 6 & 8	Amplification	ATP8.2	5’ AAA GCR TYR GCC TTT TAA GC 3’
		CO3.2	5’ GTT AGT GGT CAK GGG CTT GGR TC 3’
	Sequencing	ATP8.2	5’ AAA GCR TYR GCC TTT TAA GC 3’
RAG1	Amplification	TriRag1-F	5’ GTG TCC AGC TCY TGG AAT GT 3’
		TriRag1-R	5’ GGC ACA TGR GCC AGT GTC TTG 3’
	Sequencing	TriRag1-iF	5’ GCA CAG GCT ATG ATG AGA AGA 3’
		TriRag1-iR	5’ ATC TCA TAR CGC TCA AGG TTT TC 3’
COI	Amplification and sequencing	SilCOI-D	5' GGA GCT ACA ATC CRC CGC C 3'
		SilCOI-R	5' GGR ATK GCR ATA ATT ATT GTT GC 3'
