# Supplementary material for: Multifactorial genetic divergence processes drive the onset of speciation in an Amazonian fish
Source: PLoS One. 2017 Dec 20;12(12):e0189349. doi: 10.1371/journal.pone.0189349 (PMC5738069; doi:10.1371/journal.pone.0189349)
Supplement: S2 Table — BC (population from the Negro and Tapajós rivers, black- and clearwater rivers); CA (population from the Central Amazon and Lower Madeira River, whitewater rivers); and UM (population from the Upper Madeira, whitewater river). The analysis was based on the barcode region (647 bp) and was conducted in Mega v. 6.06 using the Kimura 2-parameters model (K2P). (PDF) [file pone.0189349.s004.pdf]

S2 Table. Estimates of COI sequence divergence between pairs of *Triportheus* species, and between pairs of lineages of *T. albus*. BC (population from the Negro and Tapajós rivers, black- and clearwater rivers); CA (population from the Central Amazon and Lower Madeira River, whitewater rivers); and UM (population from the Upper Madeira, whitewater river). The analysis was based on the barcode region (647 bp) and was conducted in Mega v. 6.06 using the Kimura 2-parameter model (K2P).

		Tang	Tgue	Tnem	Taur	Tbra	Talb		
							BC	CA	UM
T. angulatus (Tang)		*							
T. guentheri (Tgue)		0.081	*						
T. nematurus (Tnem)		0.049	0.093	*					
T. auritus (Taur)		0.168	0.170	0.179	*				
T. brachipomus (Tbra)		0.152	0.175	0.155	0.102	*			
T. albus (Talb)	BC	0.115	0.075	0.115	0.174	0.181	*		
	CA	0.108	0.071	0.107	0.174	0.180	0.006	*	
	UM	0.108	0.071	0.107	0.174	0.180	0.007	0.001	*
