# Supplementary material for: Multifactorial genetic divergence processes drive the onset of speciation in an Amazonian fish
Source: PLoS One. 2017 Dec 20;12(12):e0189349. doi: 10.1371/journal.pone.0189349 (PMC5738069; doi:10.1371/journal.pone.0189349)
Supplement: S3 Table — (PDF) [file pone.0189349.s005.pdf]

Table S3. Haplotypes of ATPase6&8.

Species	Haplotype	Accession number	Locality	N	Source	Haplotype according to the source
T. albus	Hap 1	JQ622254	a1	2	(Cooke et al. 2012)	TA1
T. albus	Hap 1	JQ622254	a2	17	(Cooke et al. 2012)	TA1
T. albus	Hap 1	JQ622254	a3	11	(Cooke et al. 2012)	TA1
T. albus	Hap 1	JQ622254	a4	3	(Cooke et al. 2012)	TA1
T. albus	Hap 1	JQ622254	a5	1	(Cooke et al. 2012)	TA1
T. albus	Hap 1	JQ622254	a6	1	(Cooke et al. 2012)	TA1
T. albus	Hap 1	MF188209	aru	9	present study	
T. albus	Hap 1	JQ622254	b1	1	(Cooke et al. 2012)	TA1
T. albus	Hap 1	MF188209	ctl	17	present study	
T. albus	Hap 1	MF188209	jac	1	present study	
T. albus	Hap 1	MF188209	pur	2	present study	
T. albus	Hap 1	MF188209	sam	5	present study	
T. albus	Hap 2	JQ622275	a1	1	(Cooke et al. 2012)	TA28
T. albus	Hap 3	JQ622263	a1	2	(Cooke et al. 2012)	TA14
T. albus	Hap 3	JQ622263	a6	1	(Cooke et al. 2012)	TA14
T. albus	Hap 4	JQ622276	a1	1	(Cooke et al. 2012)	TA29
T. albus	Hap 5	JQ622277	a1	1	(Cooke et al. 2012)	TA30
T. albus	Hap 6	JQ622278	a1	1	(Cooke et al. 2012)	TA31
T. albus	Hap 7	JQ622256	a2	1	(Cooke et al. 2012)	TA3
T. albus	Hap 8	JQ622255	a2	1	(Cooke et al. 2012)	TA2
T. albus	Hap 8	JQ622255	a3	1	(Cooke et al. 2012)	TA2
T. albus	Hap 8	JQ622255	m1	1	(Cooke et al. 2012)	TA2

Species	Haplotype	Accession number	Locality	N	Source	Haplotype according to the source
T. albus	Hap 9	JQ622264	a2	1	(Cooke et al. 2012)	TA16
T. albus	Hap 10	JQ622257	a3	1	(Cooke et al. 2012)	TA4
T. albus	Hap 11	JQ622258	a3	1	(Cooke et al. 2012)	TA6
T. albus	Hap 11	JQ622258	a5	2	(Cooke et al. 2012)	TA6
T. albus	Hap 12	JQ622259	a3	1	(Cooke et al. 2012)	TA7
T. albus	Hap 13	JQ622260	a3	1	(Cooke et al. 2012)	TA8
T. albus	Hap 14	JQ622261	a3	1	(Cooke et al. 2012)	TA9
T. albus	Hap 15	JQ622262	a6	3	(Cooke et al. 2012)	TA13
T. albus	Hap 15	JQ622262	b1	3	(Cooke et al. 2012)	TA13
T. albus	Hap 15	JQ622262	n1	2	(Cooke et al. 2012)	TA13
T. albus	Hap 15	JQ622262	t1	5	(Cooke et al. 2012)	TA13
T. albus	Hap 16	MF188210	ara	3	present study	
T. albus	Hap 16	MF188210	cau	6	present study	
T. albus	Hap 16	MF188210	jac	26	present study	
T. albus	Hap 16	JQ622271	m1	1	(Cooke et al. 2012)	TA24
T. albus	Hap 16	MF188210	pur	2	present study	
T. albus	Hap 16	MF188210	slo	4	present study	
T. albus	Hap 16	MF188210	sot	16	present study	
T. albus	Hap 17	MF188211	aru	1	present study	
T. albus	Hap 18	JQ622265	b1	5	present study	TA17
T. albus	Hap 18	JQ622265	n1	1	present study	TA17
T. albus	Hap 18	JQ622265	t1	13	present study	TA17
T. albus	Hap 19	JQ622266	b1	7	present study	TA18

Species	Haplotype	Accession number	Locality	N	Source	Haplotype according to the source
T. albus	Hap 19	JQ622266	ctl	1	present study	TA18
T. albus	Hap 20	JQ622267	b1	1	present study	TA19
T. albus	Hap 21	JQ622268	b1	2	present study	TA20
T. albus	Hap 21	JQ622268	n1	3	present study	TA20
T. albus	Hap 22	JQ622269	b1	1	present study	TA21
T. albus	Hap 23	MF188212	cau	1	present study	
T. albus	Hap 24	MF188213	cau	1	present study	
T. albus	Hap 24	MF188213	sot	1	present study	
T. albus	Hap 25	MF188214	cau	1	present study	
T. albus	Hap 26	MF188215	ctl	1	present study	
T. albus	Hap 27	MF188216	ctl	1	present study	
T. albus	Hap 27	JQ622270	m1	1	present study	TA23
T. albus	Hap 28	MF188217	ctl	1	present study	
T. albus	Hap 29	MF188218	jac	1	present study	
T. albus	Hap 30	MF188219	jac	1	present study	
T. albus	Hap 31	MF188220	jac	1	present study	
T. albus	Hap 32	MF188221	jac	1	present study	
T. albus	Hap 33	MF188222	jac	1	present study	
T. albus	Hap 34	JQ622272	n1	1	present study	TA25
T. albus	Hap 35	JQ622273	n1	1	present study	TA26
T. albus	Hap 36	JQ622274	n1	1	present study	TA27
T. albus	Hap 37	MF188223	sam	1	present study	
T. albus	Hap 38	MF188224	sam	1	present study	

Species	Haplotype	Accession number	Locality	N	Source	Haplotype according to the source
T. albus	Hap 39	MF188225	sot	1	present study	
T. albus	Hap 40	JQ622279	t1	1	present study	TA32
T. albus	Hap 41	JQ622280	t1	1	present study	TA33
T. albus	Hap 42	JQ622281	t1	1	present study	TA34
T. auritus	Hap 43	MF188226	Cuniã Lake, Madeira River	7	present study	
T. auritus	Hap 43	MF188226	Madeira River	1	present study	
T. auritus	Hap 44	MF188227	Cuniã Lake, Madeira River	1	present study	
T. auritus	Hap 45	MF188228	Cuniã Lake, Madeira River	2	present study	
T. auritus	Hap 46	JQ622253	Amazon River	1	(Cooke et al. 2012)	
T. culter	Hap 47	MF188229	Karipunas River, Madeira River	1	present study	
T. brachipomus	Hap 48	MF188230	Maroni River	1	present study	
T. brachipomus	Hap 49	MF188231	Maroni Basin	1	present study	
Brycon oligolepis		AF412668	Atrato River			
