# Supplementary material for: Multifactorial genetic divergence processes drive the onset of speciation in an Amazonian fish
Source: PLoS One. 2017 Dec 20;12(12):e0189349. doi: 10.1371/journal.pone.0189349 (PMC5738069; doi:10.1371/journal.pone.0189349)
Supplement: S4 Table — (PDF) [file pone.0189349.s006.pdf]

Table S4. Haplotypes of RAG1.

Species	Haplotype	Accession number	Locality	N	Source
Triportheus albus	Hap 1	MF188235	aru	2	Present study
Triportheus albus	Hap 2	MF188236	aru	2	Present study
Triportheus albus	Hap 2	MF188236	cau	2	Present study
Triportheus albus	Hap 2	MF188236	ctl	3	Present study
Triportheus albus	Hap 2	MF188236	jac	2	Present study
Triportheus albus	Hap 2	MF188236	pur	2	Present study
Triportheus albus	Hap 2	MF188236	slo	4	Present study
Triportheus albus	Hap 2	MF188236	sot	2	Present study
Triportheus albus	Hap 3	MF188237	aru	1	Present study
Triportheus albus	Hap 4	MF188238	aru	1	Present study
Triportheus albus	Hap 5	MF188239	aru	1	Present study
Triportheus albus	Hap 6	MF188240	aru	2	Present study
Triportheus albus	Hap 6	MF188240	slo	1	Present study
Triportheus albus	Hap 6	MF188240	sot	1	Present study
Triportheus albus	Hap 7	MF350633	aru	1	Present study
Triportheus albus	Hap 7	MF350633	cau	2	Present study
Triportheus albus	Hap 7	MF350633	pur	1	Present study
Triportheus albus	Hap 8	MF350634	cau	4	Present study
Triportheus albus	Hap 8	MF350634	jac	1	Present study
Triportheus albus	Hap 8	MF350634	sot	1	Present study
Triportheus albus	Hap 9	MF350635	ctl	1	Present study
Triportheus albus	Hap 10	MF350636	ctl	1	Present study
Triportheus albus	Hap 11	MF350637	ctl	1	Present study

Triportheus albus	Hap 12	MF350638	ctl	1	Present study
Triportheus albus	Hap 13	MF350639	ctl	2	Present study
Triportheus albus	Hap 13	MF350639	t1	1	Present study
Triportheus albus	Hap 14	MF350640	ctl	1	Present study
Triportheus albus	Hap 15	MF350641	jac	1	Present study
Triportheus albus	Hap 15	MF350641	slo	1	Present study
Triportheus albus	Hap 15	MF350641	sot	1	Present study
Triportheus albus	Hap 16	MF350642	n1	6	Present study
Triportheus albus	Hap 17	MF350643	n1	1	Present study
Triportheus albus	Hap 18	MF350644	n1	1	Present study
Triportheus albus	Hap 19	MF350645	pur	1	Present study
Triportheus albus	Hap 20	MF350646	sam	4	Present study
Triportheus albus	Hap 21	MF350647	sam	2	Present study
Triportheus albus	Hap 22	MF350648	sam	2	Present study
Triportheus albus	Hap 23	MF350649	sot	2	Present study
Triportheus albus	Hap 24	MF350650	sot	1	Present study
Triportheus albus	Hap 25	MF350651	t1	1	Present study
Triportheus albus	Hap 26	MF350652	t1	1	Present study
Triportheus albus	Hap 27	MF350653	t1	1	Present study
Triportheus albus	Hap 28	MF350654	t1	1	Present study
Triportheus albus	Hap 29	MF350655	t1	1	Present study
Triportheus albus	Hap 30	MF350656	t1	1	Present study
Triportheus albus	Hap 31	MF350657	t1	1	Present study
