# Supplementary material for: Multifactorial genetic divergence processes drive the onset of speciation in an Amazonian fish
Source: PLoS One. 2017 Dec 20;12(12):e0189349. doi: 10.1371/journal.pone.0189349 (PMC5738069; doi:10.1371/journal.pone.0189349)
Supplement: S5 Table — (PDF) [file pone.0189349.s007.pdf]

Table S5. Haplotypes of COI.

Species	Haplotype	Accession number	Locality	N	Source
T. albus	Hap_1	MF182357	t1	1	Present study
T. albus	Hyp_2	MF182358	n1	1	Present study
T. albus	Hap_3	MF182359	n1	1	Present study
T. albus	Hap_4	MF182360	n1	1	Present study
T. albus	Hap_5	MF182361	t1	2	Present study
T. albus	Hap_5	MF182361	n1	1	Present study
T. albus	Hap_6	MF182362	jac	5	Present study
T. albus	Hap_6	MF182362	sot	1	Present study
T. albus	Hap_7	MF182363	sam	1	Present study
T. albus	Hap_7	MF182363	aru	2	Present study
T. albus	Hap_7	MF182363	ctl	1	Present study
T. auritus	Hap_8	MF182364	sam	1	Present study
T. brachipomus	Hap_9	MF182365	Maroni River	1	Present study
T. angulatus	-	GU060427	-	1	(Ardura et al. 2010)
T. guentheri	-	HM404957	-	1	(Carvalho et al. 2011)
T. nematurus	-	KM897633	-	1	(Frantine-Silva et al. 2015)
T. nematurus	-	KM897647	-	1	(Frantine-Silva et al. 2015)
T. nematurus	-	KM897659	-	1	(Frantine-Silva et al. 2015)
