# Supplementary material for: Multifactorial genetic divergence processes drive the onset of speciation in an Amazonian fish
Source: PLoS One. 2017 Dec 20;12(12):e0189349. doi: 10.1371/journal.pone.0189349 (PMC5738069; doi:10.1371/journal.pone.0189349)
Supplement: S6 Table — (PDF) [file pone.0189349.s008.pdf]

Table S6. Average values of pH and transparency of the water in different sites across the Amazon Basin.

Site	Code	pH	Transparency (cm)	Source	Date of sampling	Periodicity
Cautário River	cau	6.87	96.56	present work	07/2009, 09/2009 and 01/2010	–
Sotério River	sot	6.95	25.69	present work	07/2009, 09/2009 and 01/2010	–
Arara River	ara	6.96	14.71	present work	04/2009–03/2010	monthly
São Lourenço River	slo	6.86	13.07	present work	04/2009–03/2010	monthly
Jaciparaná River	jac	6.97	12.9	present work	04/2009–03/2010	monthly
Puruzinho Lake	pur	6.69	16.8	present work	04/2009–03/2010	monthly
Sampaio Lake	sam	6.69	18.29	present work	07/2009, 09/2009 and 01/2010	–
Madeira River	m1	7.1	5.5	Cooke et al. 2012	01–02/2005 and 01–02/2008	–
Amazon River 1	a1	7.2	12.3	Cooke et al. 2012	01–02/2005 and 01–02/2008	–
Amazon River 2	a2	7.1	18.8	Cooke et al. 2012	01–02/2005 and 01–02/2008	–
Amazon River 3	a3	7.1	18.3	Cooke et al. 2012	01–02/2005 and 01–02/2008	–
Amazon River 4	a4	7.2	10.5	Cooke et al. 2012	01–02/2005 and 01–02/2008	–
Amazon River 5	a5	7.2	12.5	Cooke et al. 2012	01–02/2005 and 01–02/2008	–
Amazon River 6	a6	7.2	15	Cooke et al. 2012	01–02/2005 and 01–02/2008	–
Catalão Lake	ctl	6.75	77.5	Brito et al. 2014	11/2004–08/2005	quarterly
Ariaú Channel	aru	6.21	24.56	present work	Sep-11	unique
Branco River	b1	6.91	83.4	Cooke et al. 2012	01–02/2005 and 01–02/2008	–
Negro River	n1	5.2	76	Cooke et al. 2012	01–02/2005 and 01–02/2008	–
Tocantins River	t1	6.7	118	Cooke et al. 2012	01–02/2005 and 01–02/2008	–
