# Supplementary material for: Multifactorial genetic divergence processes drive the onset of speciation in an Amazonian fish
Source: PLoS One. 2017 Dec 20;12(12):e0189349. doi: 10.1371/journal.pone.0189349 (PMC5738069; doi:10.1371/journal.pone.0189349)
Supplement: S7 Table — Total Area of the polygon (a), area of the main channel (b) and the area occupied by lateral lakes and water bodies (c). The index was calculated using this formula: [(c/a) + (c/(b+c)) + (c/b)]/3. (PDF) [file pone.0189349.s009.pdf]

S7 Table. Floodplain index. Total Area of the polygon (a), area of the main channel (b) and the area occupied by lateral lakes and water bodies (c). The index was calculated using this formula: $[(c/a) + (c/(b+c)) + (c/b)]/3$.

Site	mm-yy	Total Area (km^2)	Main channel (km^2)	Lateral water bodies (km^2)	Index
cau	Jul-04	20.165	2.5309	2.7668	0.5842
sot	Apr-14	22.719	4.7376	2.6278	0.3424
ara	Apr-13	58.588	8.0745	0.1640	0.0143
slo	Jun-10	58.921	9.4063	0.1848	0.0140
jac	Jun-10	77.876	13.7363	0.2981	0.0156
cun	Jul-02	140.738	19.3659	27.0192	0.7232
pur	May-08	299.211	42.9834	4.3188	0.0687
sam	Jan-11	235.808	31.8603	50.4140	0.8030
m1	Apr-13	454.441	59.9223	28.5716	0.2875
a2	Apr-10	1'537.215	240.2635	252.2973	0.5755
a1	Apr-10	643.631	138.4771	127.8846	0.5341
ctl	Jun-14	697.184	177.8910	111.5075	0.3907
b1	Apr-10	374.033	94.3154	60.5554	0.3983
n1	Jun-14	1'269.073	307.1935	44.2883	0.1017
a3	Apr-14	817.357	135.1613	252.0837	0.9415
a4	Oct-13	529.916	120.4932	178.3345	0.8045
a5	Feb-14	1'161.970	261.1646	384.7297	0.8000
a6	Jul-13	847.683	236.3890	290.2333	0.7071
t1	Apr-10	4'822.269	1240.6861	41.7125	0.0249
aru	–	–	–	–	0.5341
