# Supplementary material for: Multifactorial genetic divergence processes drive the onset of speciation in an Amazonian fish
Source: PLoS One. 2017 Dec 20;12(12):e0189349. doi: 10.1371/journal.pone.0189349 (PMC5738069; doi:10.1371/journal.pone.0189349)
Supplement: S8 Table — Secondary forest (SF); anthropic area (AP); open areas, including savannahs, forested campinaranas, and contact zone area between savannahs and ombrophilous forest (SCO); dense ombrophilous forest (DO); and open ombrophilous forest (OO). Original data comes from the Ministério do Meio Ambiente of the Brazilian Government (http://mapas.mma.gov.br/mapas/aplic/probio/datadownload.htm). (PDF) [file pone.0189349.s010.pdf]

Table S8. Area (in km^2) occupied by each type of vegetation around the sampling localities. Secondary forest (SF); anthropic area (AP); open areas, including savannahs, forested campinaranas, and contact zone area between savannahs and ombrophilous forest (SCO); dense ombrophilous forest (DO); and open ombrophilous forest (OO). Original data comes from the *Ministério do Meio Ambiente of the Brazilian Government* (http://mapas.mma.gov.br/mapas/aplic/probio/datadownload.htm).

Site	SF	AP	SCO	DO	OO
a1	78.34	133.79	0.00	161.51	0.00
a2	80.72	24.09	0.00	519.85	57.64
a3	408.79	122.09	213.46	823.80	183.76
a4	148.53	0.00	130.13	187.67	47.22
a5	76.86	109.66	900.62	255.79	31.00
a6	746.14	165.77	2361.01	1090.53	125.28
ara	22.29	17.17	0.00	0.00	0.00
aru	0.00	166.31	0.00	261.55	8.74
b1	13.97	4.45	729.42	625.95	84.62
cau	0.00	7.04	0.00	0.00	81.66
ctl	42.96	9.86	0.00	273.80	0.00
jac	0.00	15.61	3.07	56.56	74.42
m1	16.92	11.22	0.00	138.72	13.01
n1	78.71	1.55	6.43	339.64	0.00
pur	0.00	1.65	0.00	56.51	129.42
sam	58.07	0.10	0.00	7747.06	0.00
slo	0.00	19.33	68.01	11.99	0.00
sot	3.62	0.00	0.00	0.00	86.54
t1	619.20	99.88	0.00	2142.13	0.00
