# Supplementary figures and images for: Multifactorial genetic divergence processes drive the onset of speciation in an Amazonian fish
Source: PLoS One. 2017 Dec 20;12(12):e0189349. doi: 10.1371/journal.pone.0189349 (PMC5738069; doi:10.1371/journal.pone.0189349)

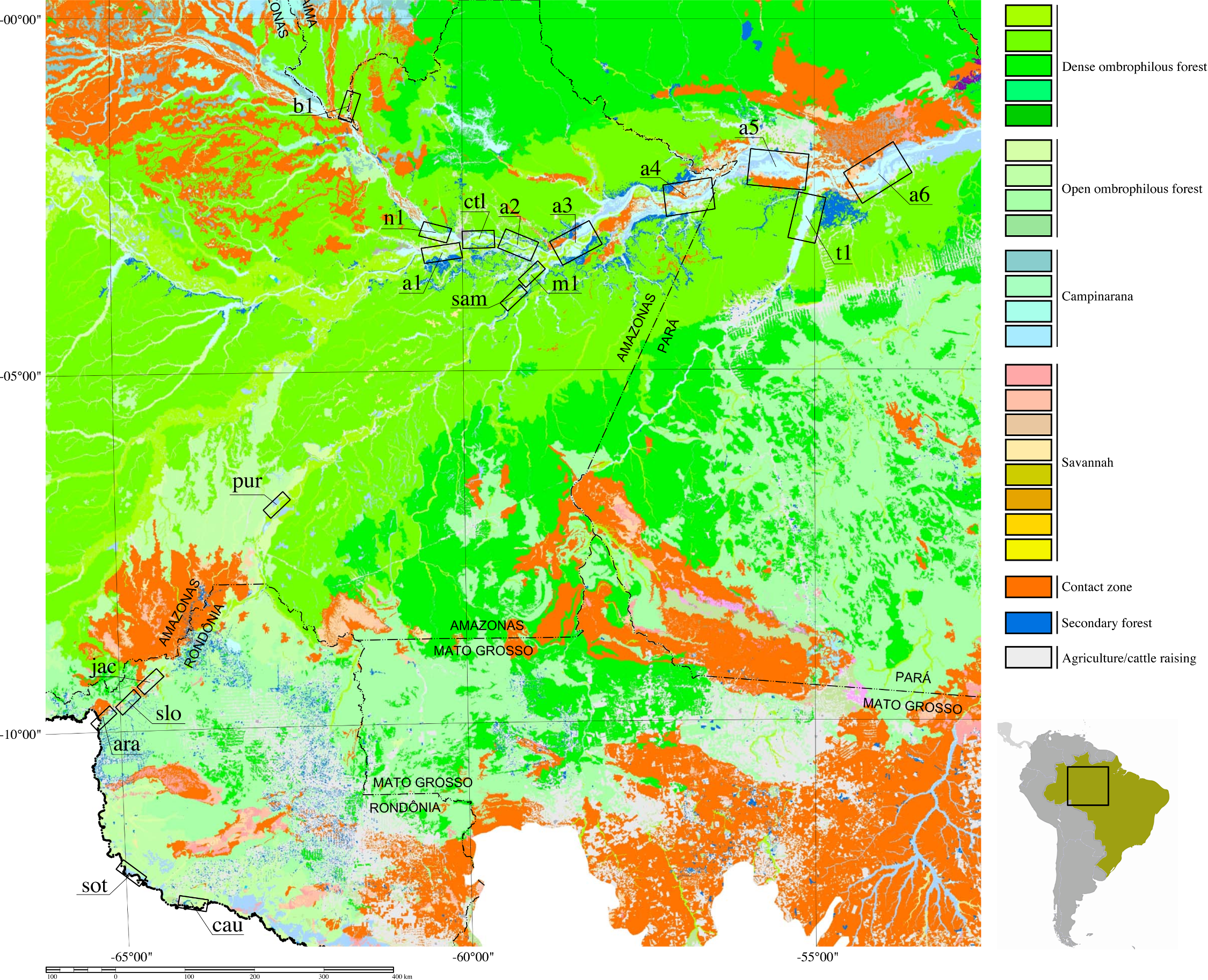

Supplement: S1 Fig — A. Maximum likelihood tree of Triportheus albus and close species. The tree shown here is based on the ATPase 6 & 8 marker. It contains a subsample of T. albus representatives of each of the three lineages: (i) in green, samples from the Upper Madeira lineage; (ii) in red, samples from the Lower Madeira and Central Amazon lineage; and (iii) in blue, samples from the black- and clearwaters of the Negro and Tapajós rivers. Representatives of three other congeneric species are included. This tree supports the monophyly of T. albus. Values in the nodes represent node support, which were estimated by 1000 bootstrap replicates. Values lower than 50 are omitted. B. As the T. albus lineages show particularly very short branch lengths, we present the same tree transformed into a cladogram. (TIFF) [file pone.0189349.s011.tiff]

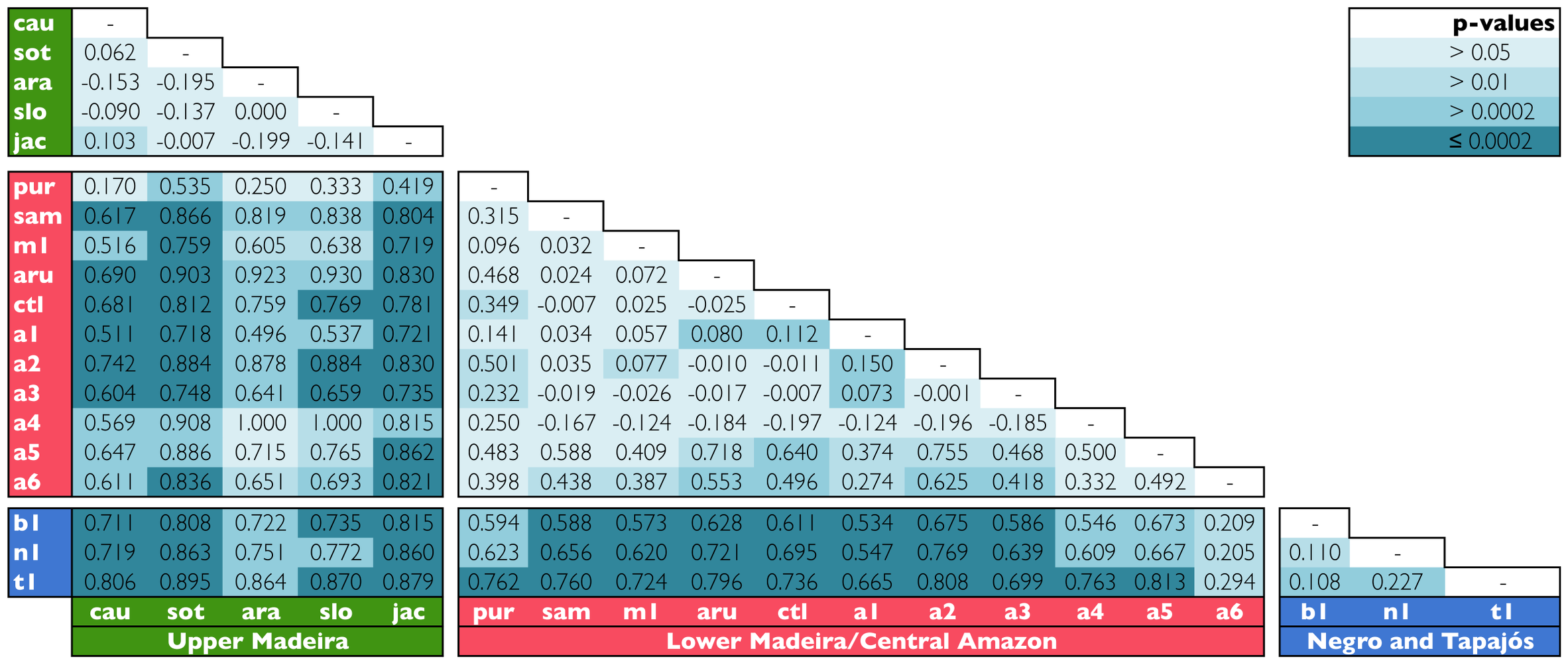

Supplement: S2 Fig — The polygons represent the area in which the proportion of the different vegetation categories was calculated for each sampling site. This map and those with higher resolution (1:250’000), which were used for a more precise estimation of the proportion of each vegetation categories, are provided by the Brazilian Government (Ministério do Meio Ambiente) and are freely available in the public domain (http://mapas.mma.gov.br/mapas/aplic/probio/datadownload.htm). Vegetation categories present in the original maps but which were not found in the areas analysed in this study were omitted in this map. (TIF) [file pone.0189349.s012.tif]

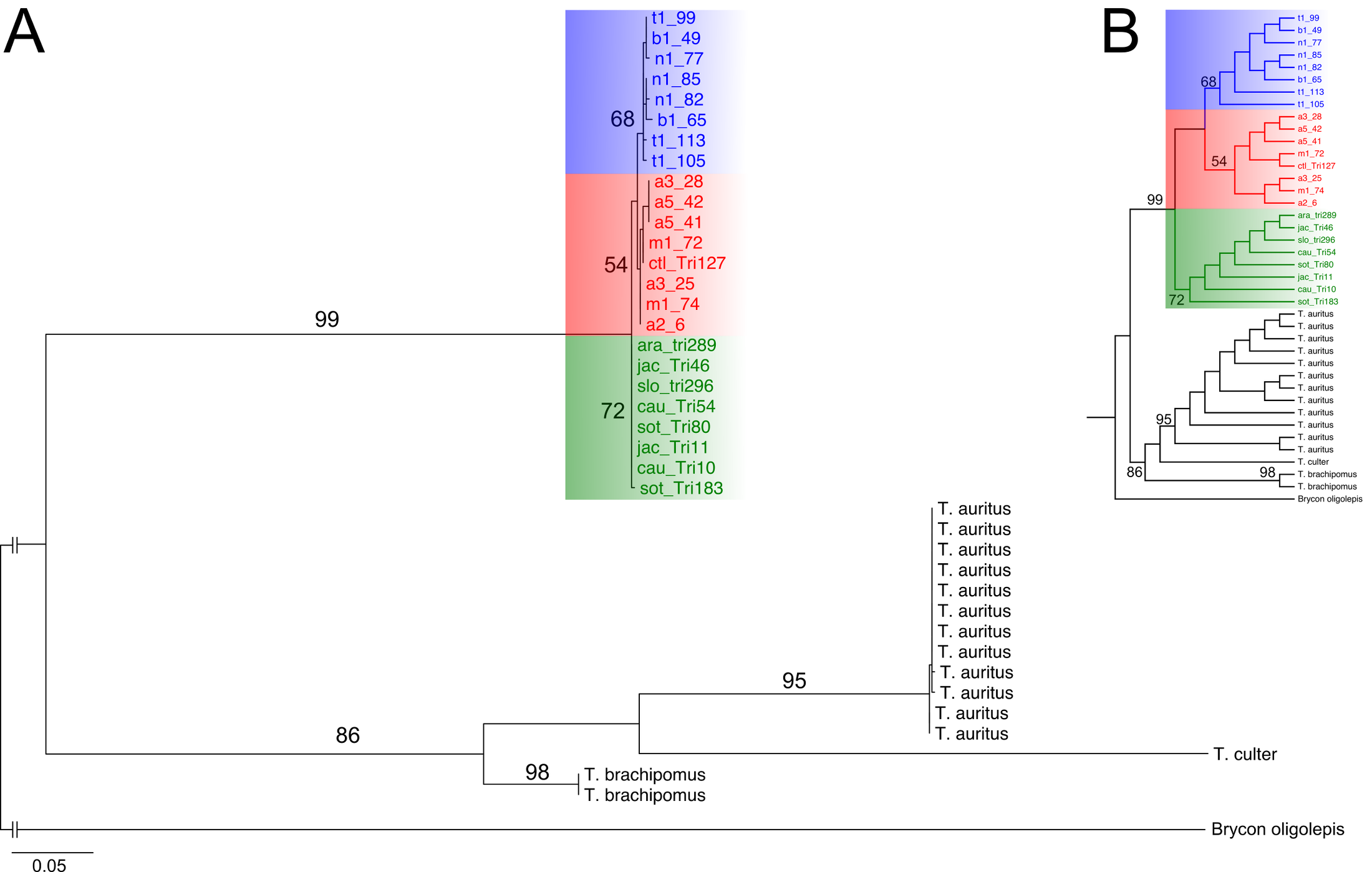

Supplement: S3 Fig — Colours intensity represents the significance level. (TIFF) [file pone.0189349.s013.tiff]
